# The midnolin-proteasome pathway catches proteins for ubiquitination-independent degradation

**DOI:** 10.1126/science.adh5021

**Published:** 2023-08-25

**Authors:** Xin Gu, Christopher Nardone, Nolan Kamitaki, Aoyue Mao, Stephen J. Elledge, Michael E. Greenberg

**Affiliations:** 1 Department of Neurobiology, Harvard Medical School, Boston, MA 02115, USA; 2 Division of Genetics, Department of Medicine, Howard Hughes Medical Institute, Brigham and Women’s Hospital, Boston, MA 02115, USA; 3 Department of Genetics, Harvard Medical School, Boston, MA 02115, USA; 4 Department of Biomedical Informatics, Harvard Medical School, Boston, MA 02115, USA; 5 Department of Molecular and Cellular Biology, Harvard University, Cambridge, MA 02138, USA

## Abstract

Cells use ubiquitin to mark proteins for proteasomal degradation. While the proteasome also eliminates proteins that are not ubiquitinated, how this occurs mechanistically is unclear. Here we found that midnolin promoted the destruction of many nuclear proteins including transcription factors encoded by the immediate-early-genes. Diverse stimuli induced midnolin and its overexpression was sufficient to cause the degradation of its targets by a mechanism that did not require ubiquitination. Instead, midnolin associated with the proteasome via an α helix, employed its Catch domain to bind a region within substrates that can form a β strand, and used a ubiquitin-like domain to promote substrate destruction. Thus, midnolin contains three regions that function in concert to target a large set of nuclear proteins to the proteasome for degradation.

## One-sentence summary

Midnolin associates with the proteasome to promote the degradation of many nuclear proteins via a ubiquitination-independent mechanism.

In mammals, extracellular growth factors, cytokines, neurotrophic factors, and neurotransmitters bind their cognate receptors and activate rapid responses by inducing post-translational modifications of pre-existing proteins. More delayed responses are also induced by stimulating gene transcription within the nucleus (1, 2). This transcriptional response occurs in two steps. First, within minutes of the initial stimulus, a set of genes termed immediate-early-genes (IEGs) is activated. The IEGs encode transcription factors that then trigger a second wave of late-response-gene (LRG) expression that mediates the cellular response to the initial stimulus. The IEG response is activated in a stereotypical fashion in virtually every cell type in the body but induces programs of LRG expression that are cell-type specific (3). The plethora of cellular responses regulated by IEGs include the cell cycle re-entry of quiescent fibroblasts during wound healing, the activation of immune cells in response to cytokines, bacterial, and viral infections, and the adaptive responses of neurons to neurotransmitters during learning and memory (3).

The IEG family encodes transcription factors such as *Fos, Egr,* and *Nr4a,* that are rapidly and transiently induced in response to a wide range of extracellular stimuli (4). The IEG mRNAs accumulate to a high level, and once these mRNAs are translated, the IEG proteins undergo rapid decay (5). Thus, the protein stability of the IEG program is tightly controlled to allow for a relatively brief burst of protein expression that is crucial for appropriate cellular responses to various stimuli. Mis-regulation of the signaling networks that control IEG expression can lead to cancer, immune deficiencies, and neurological disorders (3).

While the mechanisms that regulate IEG transcription are well characterized, it is unclear how IEG proteins are degraded. In many cases, conjugation of ubiquitin onto substrate proteins occurs as a prelude to their destruction by the proteasome. c-Fos and FosB have been reported to be targeted to the proteasome by both ubiquitination-dependent and -independent mechanisms, but the molecular events that orchestrate these processes are unknown (6, 7).

## Genetic screens reveal midnolin as a regulator of IEG protein degradation

**Fig. 1.**
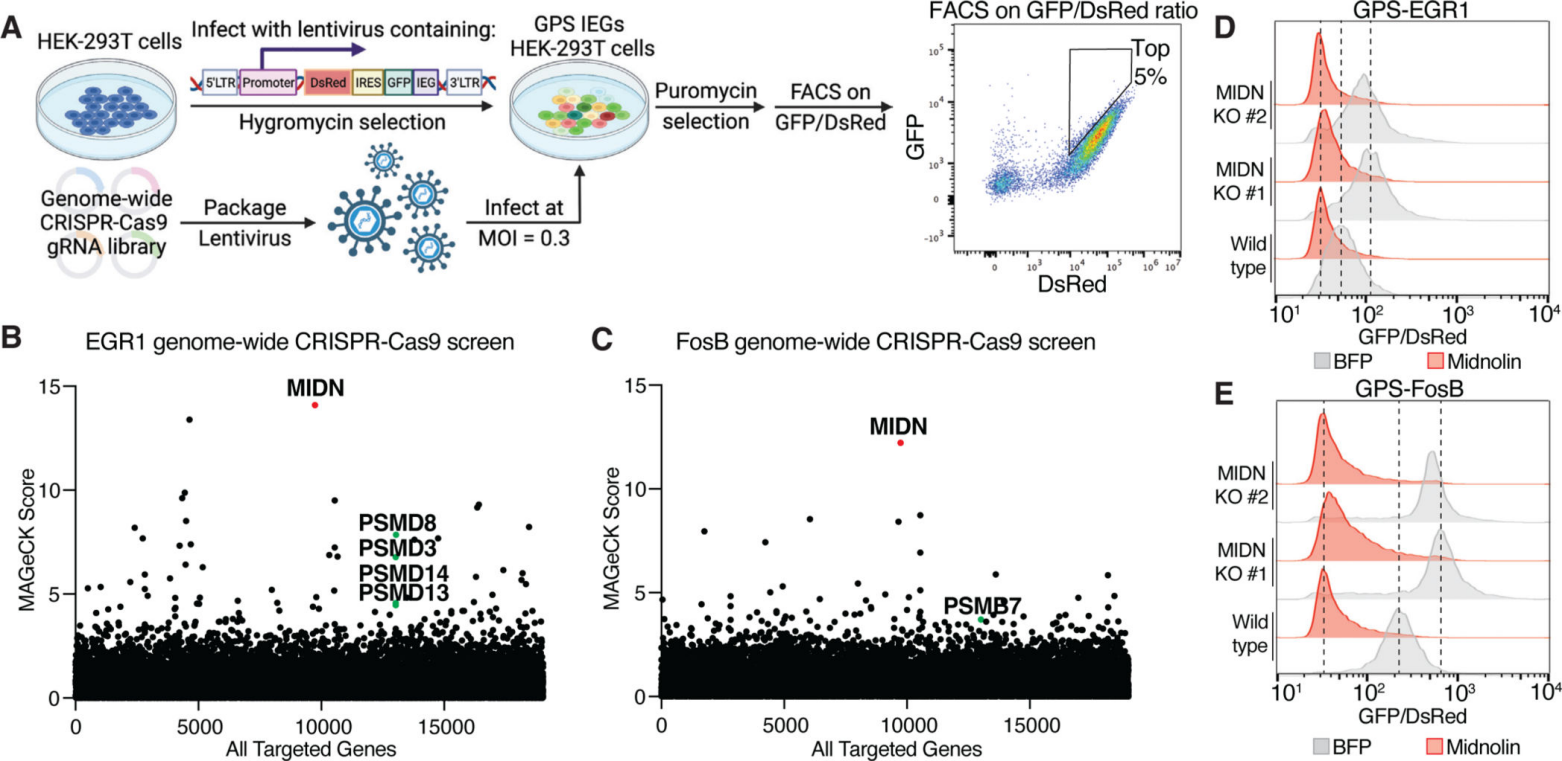
Genetic screens reveal midnolin as a regulator of IEG protein degradation. (**A**) Schematic showing the FACS-based genome-wide CRISPR-Cas9 screens using the Global Protein Stability (GPS) reporter of IEG proteins in HEK-293T cells (created with BioRender.com). (**B** and **C**) Results of the genetic screens revealed *MIDN* as the top hit for negatively regulating the stability of both EGR1 and FosB. The proteasomal components showed a weaker enrichment likely due to their essentiality. The MAGeCK score represents the negative log10 of the “pos|score” value generated from MAGeCK (56). (**D** and **E**) Losing midnolin stabilized, while overexpressing midnolin destabilized EGR1 and FosB. GPS EGR1 or FosB reporters were stably expressed in wild-type and two independent *MIDN* KO HEK-293T single cell clones. Vectors expressing BFP control alone (grey) or midnolin and BFP from a EF1α promoter (red) was transiently reintroduced by transfection before analyzing the GFP/DsRed ratio by flow cytometry.

To investigate the mechanism of IEG protein degradation, we first employed the Global Protein Stability (GPS) reporter system to assay IEG protein stability (8). GPS allows for the stable expression of DsRed as an internal control and a GFP-tagged protein from the same bicistronic mRNA. Thus, the ratio of GFP/DsRed analyzed by flow cytometry provides a measure of the relative stability of the GFP-fused protein. To identify regulators of IEG protein stability, we generated HEK-293T cell lines stably expressing the GPS reporter for EGR1 or FosB and performed genome-wide CRISPR-Cas9 screens to hunt for genes whose disruption stabilized EGR1 or FosB (Fig. 1A). The comparison of EGR1 and FosB allowed us to investigate whether IEGs from different families are degraded by same or different mechanisms. The top hit from both screens was *MIDN*, a gene that in mammals encodes a largely uncharacterized protein named midnolin (Fig. 1, B and C, and Data S1).

To validate our CRISPR-Cas9 screening results, we generated *MIDN* clonal knockout (KO) HEK-293T cells stably expressing the GPS reporter for EGR1 or FosB, and assessed the effect of *MIDN* disruption and overexpression on the stability of EGR1 or FosB. Consistent with the screening results, loss of midnolin increased the stability of both EGR1 and FosB (Fig. 1, D and E). We tested additional IEG proteins, and midnolin overexpression was sufficient to decrease the levels of EGR1, FosB, c-Fos, and NR4A1 (Fig. 1, D and E, fig. S1, A and B), but not several other transcription factors including ATF2, CREB3, and CREB5 (fig. S1C). These findings raised an intriguing possibility that three distinct families of IEG proteins may be targeted for degradation by the same protein, midnolin.

## Midnolin is induced and promotes the degradation of several IEG proteins in physiological settings

**Fig. 2.**
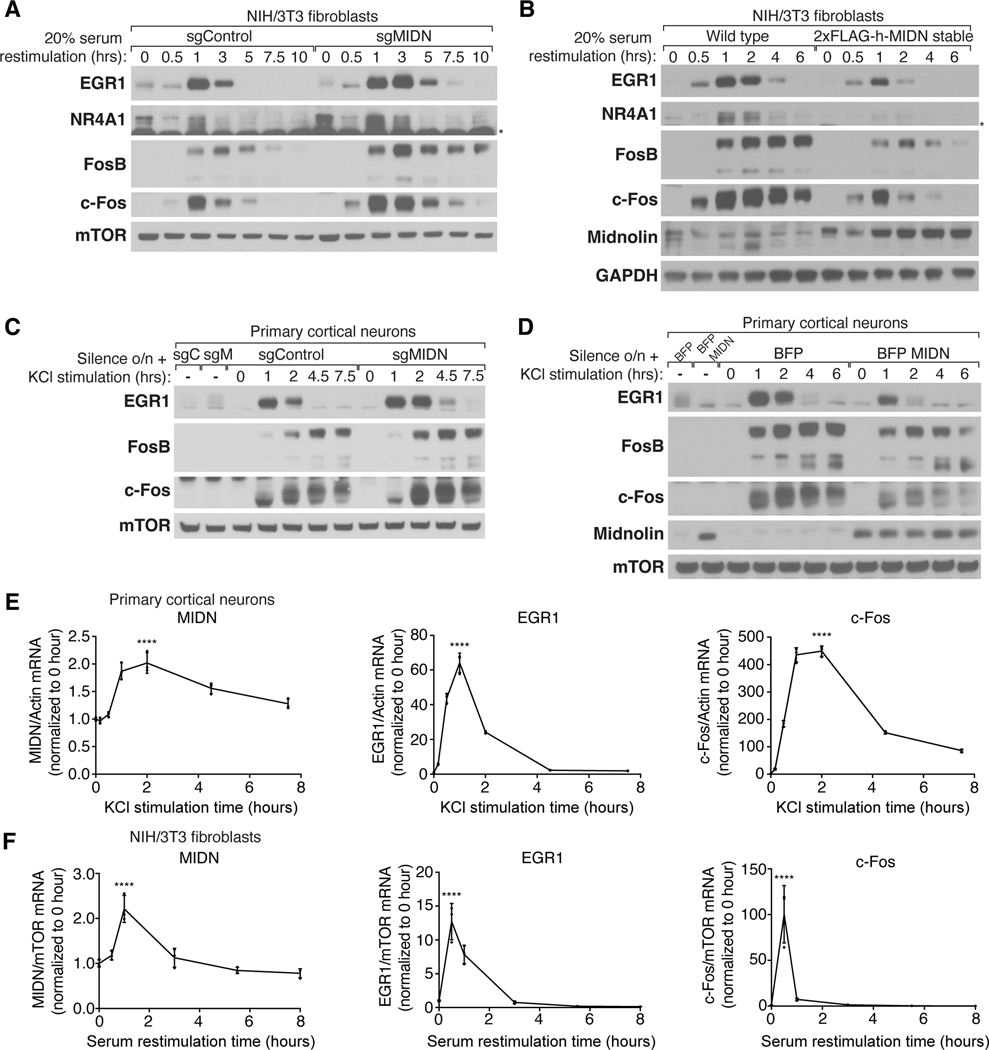
Midnolin is induced and promotes the degradation of several IEG proteins in physiological settings. (**A**) Loss of midnolin increased the expression of IEG proteins in NIH/3T3 cells. Immunoblotting was performed from NIH/3T3 cells stably expressing Cas9 and control or *MIDN* targeting single guide RNAs. This population-level mutagenesis of *MIDN* may show lower penetrance relative to an isogenic mutant since the knockout efficiency is dependent on the efficacy of the single guide RNA. The cells were starved of serum overnight before serum restimulation for the indicated time points. Asterisks mark non-specific cross-reactive proteins. (**B**) Overexpressing midnolin decreased the expression of IEG proteins in NIH/3T3 cells. Same assay as (A) but in NIH/3T3 cells stably overexpressing an N-terminally 2xFLAG tagged human midnolin using a CMV promoter. (**C**) Loss of midnolin increased the expression of IEG proteins in primary cortical neurons. Neurons were isolated from E16.5 mouse brains and cultured in a dish. On day 3 post-isolation, the neurons were infected with lentivirus encoding Cas9 with control or *MIDN* targeting single guide RNAs. Immunoblotting was performed on day 11 post dissection from neurons that were silenced overnight with tetrodotoxin (TTX, a sodium channel blocker) and D-AP5 (a NMDA receptor antagonist) and stimulated with KCl for the indicated time points to induce depolarization. (**D**) Overexpressing midnolin decreased the expression of IEG proteins in primary cortical neurons. Similar assay as (C) but using lentivirus to overexpress a BFP control or human midnolin co-expressing BFP using an EF1α promoter. (**E** and **F**) qPCR analysis for mRNA levels of the indicated genes from (E) primary mouse cortical neurons that were KCl stimulated or (F) from NIH/3T3 cells that were serum restimulated for the indicated time points. Error bars represent the standard deviation from three biological replicates. Data were analyzed using an ordinary one-way ANOVA followed by Tukey’s multiple comparisons test where **** represents p < 0.0001.

To investigate the requirement of midnolin for the degradation of IEG proteins in physiologically relevant settings, we mutated *MIDN* using CRISPR-Cas9 to generate a population-level KO of NIH/3T3 fibroblasts, a well-characterized cell line for studying IEG inducibility during cell cycle re-entry. Serum deprivation synchronizes NIH/3T3 cells in the G0 phase of the cell cycle, and IEGs are rapidly and transiently transcribed within minutes after serum addition to these cells (9). This transient induction of IEG transcription is followed by an increase in the levels of IEG proteins that then quickly return to their basal levels. In *MIDN* KO cells, IEG protein levels remained high for several hours longer than in control cells, suggesting that the degradation of EGR1, NR4A1, FosB, and c-Fos was attenuated in the absence of midnolin (Fig. 2A). In contrast, stable overexpression of midnolin led to a decrease in the level of these IEG proteins (Fig. 2B).

In addition to their importance during cell cycle re-entry, IEGs mediate adaptive responses in neurons. In response to sensory stimuli, if enough glutamate is released at excitatory synapses in the brain to generate an action potential in the post-synaptic neuron, the depolarization of the post-synaptic neuron results in an influx of calcium that triggers the induction of IEGs (3). This membrane depolarization induction of IEGs also occurs in cultured mouse neurons within minutes of exposure to elevated levels of KCl (10). To determine if midnolin regulates IEG protein levels in this paradigm, embryonic mouse cortical neurons were cultured and infected with lentivirus to generate population-level *MIDN* KO or midnolin-overexpressing cells. When exposed to elevated levels of KCl, IEG protein expression was increased in *MIDN* KO neurons but decreased in the midnolin-overexpressing neurons (Fig. 2, C and D). In contrast, IEG mRNA expression was largely unchanged by midnolin overexpression (fig. S1D), which is consistent with midnolin affecting IEG protein stability, but not their mRNA transcripts.

KCl treatment of primary cortical neurons induced *MIDN* mRNA levels with kinetics similar to that of IEGs (Fig. 2E). In a previous RNAseq dataset, *MIDN* was found to be induced upon light stimulation of the visual cortex in vivo (fig. S1E) (11). Midnolin was also induced upon serum restimulation of NIH/3T3 cells (Fig. 2F) with kinetics similar to that of IEGs (12). These stimulus-dependent increases in midnolin expression may be involved in the rapid degradation of IEG proteins. Thus, midnolin is induced by various stimuli and promotes the degradation of IEG proteins in physiologically relevant settings, potentially through a feedback mechanism.

## Midnolin can promote the degradation of numerous transcriptional regulators

**Fig. 3.**
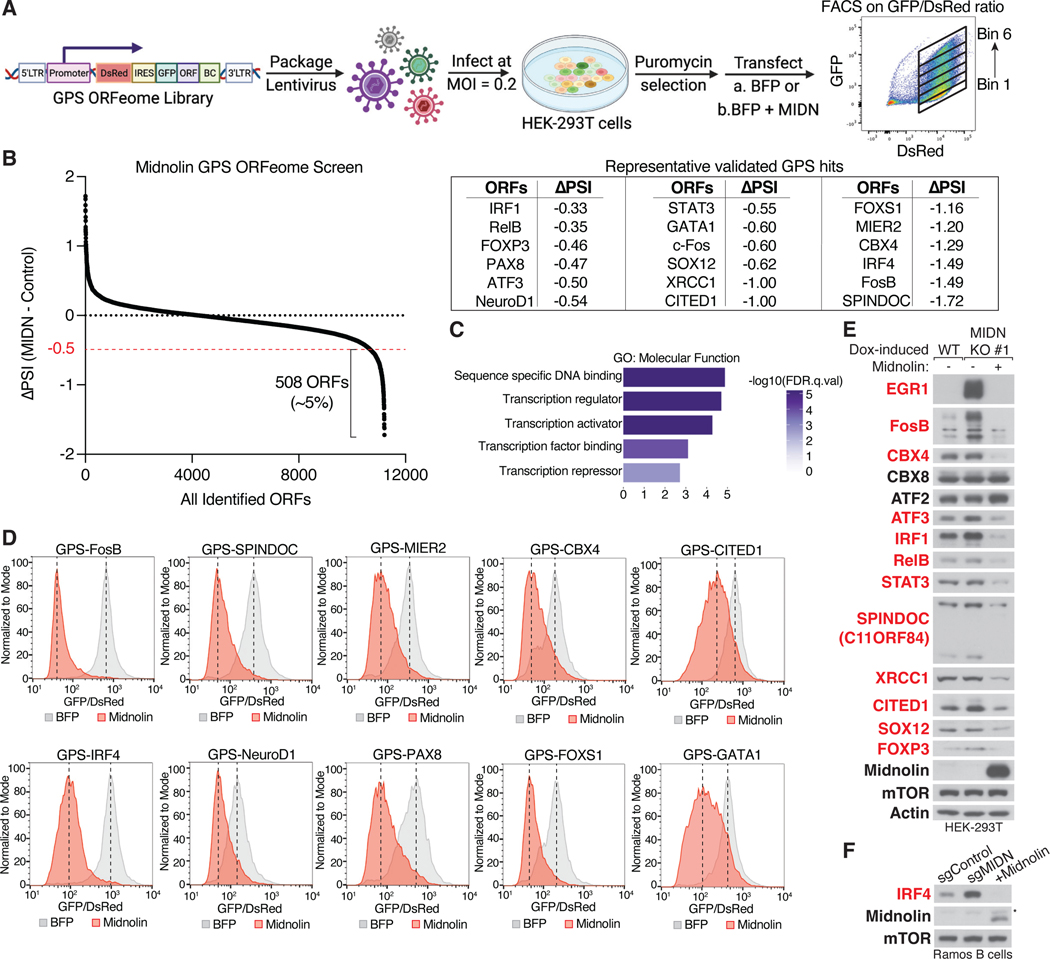
Midnolin can promote the degradation of numerous transcriptional regulators. (**A**) Schematic showing the midnolin GPS ORFeome screen. The GPS ORFeome library (~12,000 barcoded human ORFs tagged to GFP) was introduced into *MIDN* KO HEK-293T and the library-expressing cells were transfected with BFP control or midnolin co-expressing BFP before FACS sorting the library into populations based on the GFP/DsRed ratio (created with BioRender.com). (**B**) Analysis of the GPS ORFeome screen showing the change in protein stability (ΔPSI) between midnolin and BFP, which was calculated based on the change in read distribution of the barcoded ORFs. Approximately 5% of the library showed significant destabilization with ΔPSI values less than −0.5. Several validated hits from the screen are shown in the boxed table. (**C**) Gene set enrichment analysis (GSEA) based on the GPS ORFeome screen for molecular function. (**D**) Validation of screen hits indicates their potent regulation by midnolin. GPS reporters for the indicated genes were stably expressed in *MIDN* KO HEK-293T cells and a control BFP or midnolin co-expressing BFP were transiently transfected before analyzing the GFP/DsRed ratio by flow cytometry. (**E**) Endogenous proteins of numerous screen hits are regulated by midnolin. Immunoblotting was performed from wild-type, *MIDN* KO, and *MIDN* KO HEK-293T cells where midnolin expression was stably induced with doxycycline (100 ng/mL) for 2 days using a TRE promoter. Shown are putative midnolin targets (red) based on the GPS ORFeome screen and negative controls (black). (**F**) Validation of midnolin-mediated degradation of endogenous IRF4 in Ramos B cells. Immunoblotting was performed from Ramos B cells expressing Cas9 and control or *MIDN* targeting single guide RNAs, or stably overexpressing midnolin using an EF1α promoter.

To determine the extent to which midnolin regulates other cellular proteins beyond IEGs, we performed a screen to identify additional midnolin targets. The screen employed a previously described GPS ORFeome library, which contains ~12,000 barcoded human open reading frames (ORFs) tagged with GFP in the GPS reporter system (13). The GPS ORFeome library was stably introduced into *MIDN* KO HEK-293T cells and plasmids expressing either a control blue fluorescent protein (BFP), or midnolin together with BFP, were transiently transfected into cells to yield two cell libraries, one lacking midnolin and the other overexpressing midnolin. Because the GPS system overexpresses proteins and endogenous midnolin levels are low, we overproduced midnolin to gain sensitivity in this setting. The cells in each library were then partitioned into six populations based on their GFP/DsRed ratios by fluorescence-activated cell sorting. The barcodes present in each population were then sequenced to determine the change in their distribution within the cell populations upon midnolin overexpression. If midnolin promoted the destruction of a given barcoded GFP-fusion protein, the distribution of the barcode would shift to a cell population with a lower GFP/DsRed ratio in the midnolin overexpressing library (Fig. 3A). This screen yielded our previously characterized targets of midnolin including FosB and c-Fos, along with CBX4 (Pc in flies), which was previously shown to be regulated by the *Drosophila melanogaster* ortholog of midnolin, Stuxnet (14) (Fig. 3B). Midnolin overexpression also led to a robust reduction in the GFP/DsRed ratios of many proteins, consistent with a decrease in protein stability (Data S2). Most of the proteins regulated by midnolin overexpression were nuclear proteins that regulate transcription (Fig. 3C), such as the lineage-specific transcription factors IRF4, NeuroD1, PAX8, and GATA1.

To validate the findings from the GPS ORFeome screen, midnolin was overexpressed in *MIDN* KO HEK-293T cells stably expressing individual GPS reporters for the identified proteins. Midnolin overexpression was sufficient to reduce the GFP/DsRed ratios of these proteins, consistent with a decrease in their stability (Fig. 3D and fig. S2A). The endogenous levels of various proteins identified in the screen were also substantially reduced in HEK-293T cells that expressed a doxycycline-induced midnolin (Fig. 3E). Among the lineage-specific transcription factors that are not expressed in HEK-293T cells, interferon regulatory factor 4 (IRF4) is essential for the function and homeostasis of mature B and T cell lymphocytes (15, 16). To test the ability of midnolin to promote the destruction of endogenous IRF4, we generated Ramos B cell lines with population-level KO or overexpression of midnolin. In these cell lines, the steady-state levels of IRF4 were markedly increased in the *MIDN* KO cells and decreased in the midnolin-overexpressed cells (Fig. 3F). Thus, through gain-of-function screening, we uncovered many potential targets of midnolin that have important tissue or cell-type specific functions in regulating gene expression.

## Midnolin associates both with its substrates and the 26S proteasome

**Fig. 4.**
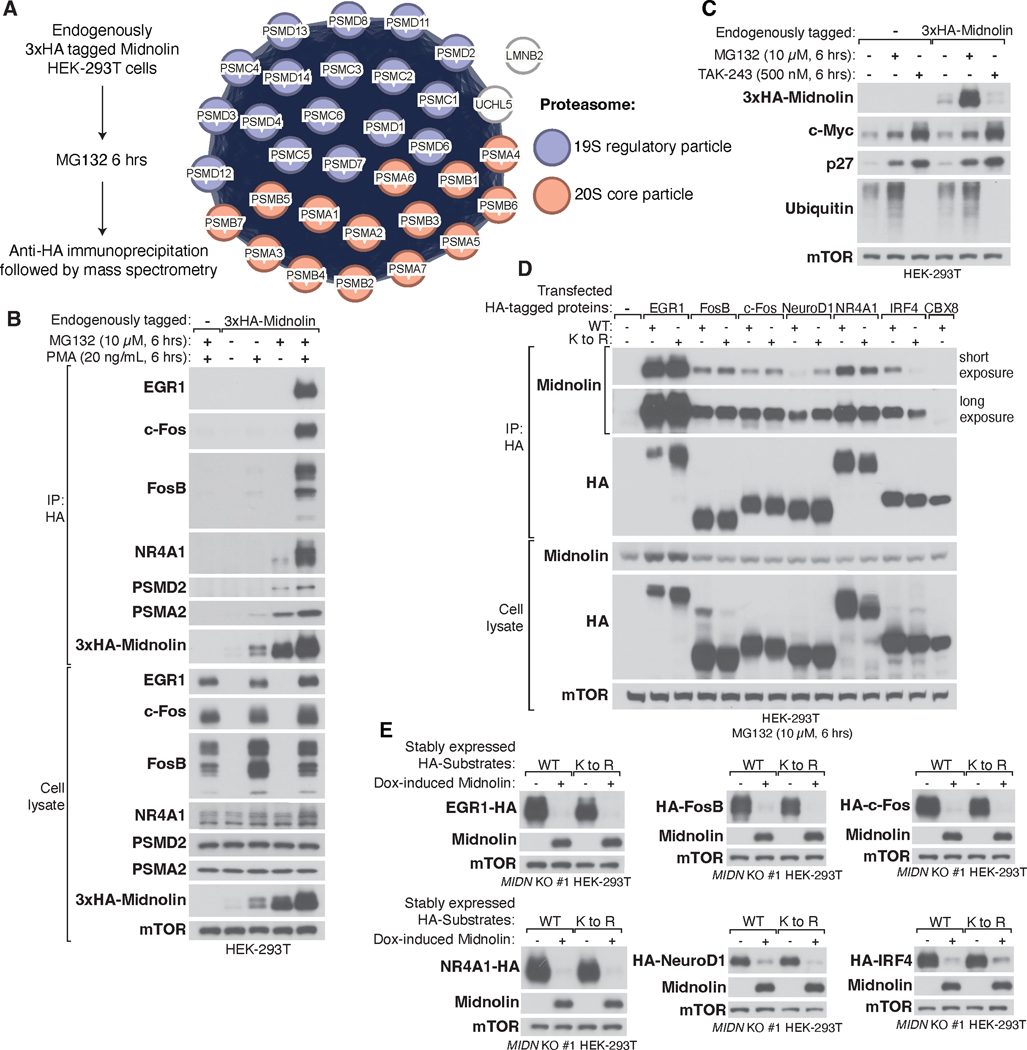
Midnolin associates with the proteasome to promote ubiquitination-independent degradation of bound substrates. (**A**) A 3xHA-tag was introduced at the N-terminus of the endogenous midnolin locus in HEK-293T cells using CRISPR-Cas9 initiated recombination. Cells were treated with MG132 for 6 hours before immunoprecipitation of 3xHA-midnolin followed by mass spectrometry. The results revealed a large enrichment of the 26S proteasome (Data S3) and shown is a STRING analysis of the top co-immunoprecipitated proteins identified from the mass spectrometry. (**B**) Midnolin co-immunoprecipitates the proteasome and IEG proteins endogenously. Immunoblotting was performed from anti-HA immunoprecipitants of endogenous 3xHA-midnolin from the knock-in HEK-293T cells treated with the indicated drugs for 6 hours. PMA was used to induce the transcription of IEGs. (**C**) Endogenous midnolin protein levels are strongly increased by proteasomal inhibition but not by ubiquitin E1 inhibition. Immunoblotting was performed from wild-type and 3xHA-midnolin knock-in HEK-293T cells treated with 10 μM MG132 or 500 nM TAK-243 for 6 hours. (**D**) Lysine-dependent ubiquitination on substrates is not necessary for midnolin interaction. Immunoblotting was performed from anti-HA immunoprecipitants of HEK-293T cells that were transfected with the indicated constructs, either wild-type or all lysine residues mutated to arginine residues (K to R). Cells were treated with 10 μM MG132 for 6 hours. CBX8 serves as a negative control as it is not targeted by midnolin. (**E**) Midnolin does not require lysine residues on substrates to promote degradation. Wild-type and K to R mutant substrates were stably introduced into *MIDN* KO HEK-293T cells using a CMV promoter. Then, midnolin expression was induced using doxycycline (100 ng/mL) for 2 days using a TRE promoter before lysis and immunoblotting.

To begin to determine how midnolin promotes the degradation of a large diverse set of proteins, we generated a HEK-293T cell line in which endogenous midnolin was tagged at its N-terminus with 3xHA to facilitate immunoprecipitation. We found by mass spectrometry that endogenous midnolin co-immunoprecipitated essentially all proteasomal subunits of the 19S regulatory particle and the 20S proteolytic core particle (Fig. 4A and Data S3). These proteasomal subunits were the most abundant proteins detected by mass spectrometry, and there were no proteins besides the proteasomal proteins that were co-immunoprecipitated by midnolin and found to be shared with the genome-wide CRISPR-Cas9 screens (Data S3 and Fig. 1, A, B, and C). These results suggested that midnolin likely interacts directly with the proteasome. In addition, by mining the BioPlex protein-protein interaction dataset (17), we found that several IEG proteins including EGR1, FosB, and NR4A1, as well as a proteasomal component, PSMD2, co-immunoprecipitated endogenous midnolin in HEK-293T cells (fig. S3A).

To confirm the mass spectrometry findings, we treated HEK-293T cells expressing 3xHA-tagged midnolin with MG132 to prevent substrate degradation or with phorbol 12-myristate 13-acetate (PMA), a Protein Kinase C (PKC) agonist to induce the transcription of IEGs (18). Endogenous midnolin interacted with c-Fos, FosB, EGR1, and NR4A1, as well as the proteasome, as indicated by PSMD2 and PSMA2, components of the 19S and 20S proteasome, respectively (Fig. 4B). Like serum-stimulated fibroblasts and KCl-treated neurons, exposure of HEK-293T cells to PMA led to an increase in the level of the midnolin protein (Fig. 4B). Thus, midnolin interacts with the proteasome to promote the degradation of midnolin-bound proteins.

## Midnolin promotes ubiquitination-independent degradation of bound substrates

We next investigated whether midnolin targets its substrates for destruction by a ubiquitination-and proteasome-dependent mechanism. Individual GPS reporters of midnolin substrates were stably expressed in *MIDN* KO HEK-293T cells, which were transfected with BFP control or midnolin co-expressing BFP. These reporter cells were then treated for 6 hours with the proteasome inhibitor MG132 or TAK-243, a potent inhibitor of the E1 ubiquitin-activating enzymes UBA6 and UAE that inhibits protein ubiquitination globally (19). While the proteasome inhibitor MG132 strongly reduced the midnolin-mediated degradation of these midnolin substrates, the ubiquitin E1 inhibitor TAK-243 did not disrupt midnolin function (fig. S3B). In contrast, both MG132 and TAK-243 promoted stabilization of c-Myc, which does not appear to be a midnolin substrate (fig. S3B). Furthermore, when we treated HEK-293T cells expressing 3xHA-tagged midnolin with MG132 or TAK-243 for 6 hours, we found that the proteasome inhibitor MG132 led to a significant increase in the expression of the midnolin protein, but the E1 inhibitor TAK-243 caused a slight decrease in the level of midnolin. In contrast, the level of c-Myc and p27, two transcriptional regulators that are known to be targeted for proteasomal degradation in a ubiquitination-dependent manner increased upon exposure to TAK-243 or MG132 (Fig. 4C) (20–22). Thus, midnolin does not appear to require ubiquitination for its own turnover by the proteasome.

To further test the requirement of ubiquitination for midnolin-mediated degradation, potential ubiquitination sites were mutated in several midnolin substrates. Because canonical ubiquitination occurs on lysine residues, all lysine residues within these substrates were mutagenized to arginine to block lysine-dependent ubiquitination (23–25). Wild-type and K to R mutant substrates, such as EGR1, FosB, c-Fos, NR4A1, NeuroD1, and IRF4, interacted with endogenous midnolin to a similar extent (Fig. 4D). When stably expressed in *MIDN* KO HEK-293T cells, both wild-type and K to R mutant substrates were efficiently degraded upon doxycycline-induced midnolin overexpression (Fig. 4E), indicating that lysine residues were not required for midnolin-dependent substrate destruction. Thus, it seems that midnolin directly associates with the proteasome and promotes the degradation of many transcriptional regulators without requiring their ubiquitination.

## Midnolin contains three domains that function in concert to promote proteasomal degradation of bound substrates

**Fig. 5.**
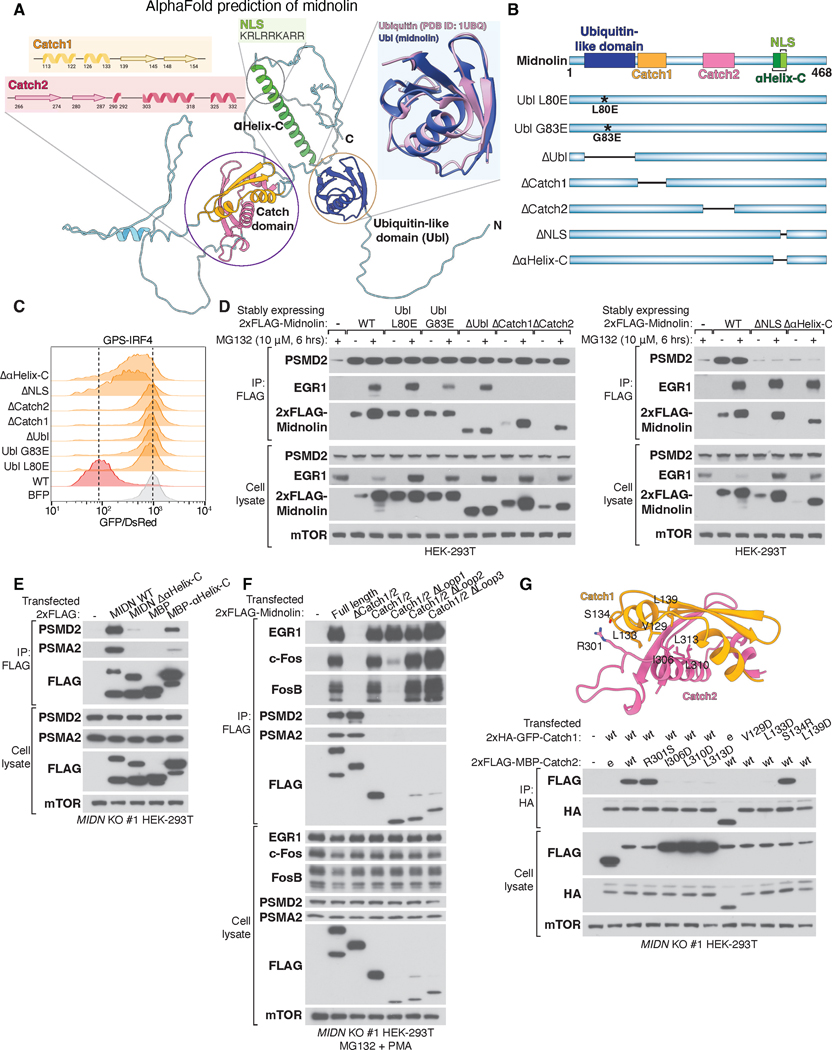
Midnolin contains three regions that function in concert to promote proteasomal degradation of bound substrates. (**A**) Midnolin structure prediction by AlphaFold (Q504T8-F1) reveals three regions with defined structure (26). (**B**) Schematic representation of mutations (57) or truncations introduced into the midnolin cDNA. See methods for the truncation boundaries and regions used for sufficiency experiments. (**C**) Regions with defined structure are necessary for a functional midnolin. The GPS IRF4 reporter was stably expressed in *MIDN* KO HEK-293T cells and a control BFP or wild-type and mutant versions of midnolin co-expressing BFP were transiently transfected before analyzing the GFP/DsRed ratio by flow cytometry. (**D**) The midnolin Catch domain is necessary for binding substrates and the C-terminal α helix is necessary for proteasomal association. Immunoblotting was performed from anti-FLAG immunoprecipitants of HEK-293T cells stably expressing 2xFLAG-tagged midnolin using a CMV promoter. Cells were treated with 10 μM MG132 for 6 hours. (**E**) The midnolin αHelix-C is sufficient to interact with the proteasome. Immunoblotting was performed from anti-FLAG immunoprecipitants of *MIDN* KO HEK-293T cells transfected with the indicated 2xFLAG-tagged proteins. (**F**) The midnolin Catch domain is sufficient to bind substrates. Immunoblotting was performed from anti-FLAG immunoprecipitants of *MIDN* KO HEK-293T cells transfected with the indicated 2xFLAG-tagged proteins. The 111 amino acid sequence between Catch1 and Catch2 was shortened to 10 amino acids (ΔLoop1), 16 amino acids (ΔLoop2), or 28 amino acids (ΔLoop3). Cells were treated with 10 μM MG132 and 20 ng/mL of PMA for 6 hours. (**G**) The Catch1 and Catch2 regions of midnolin interact when expressed as independent proteins. Immunoblotting was performed from anti-HA immunoprecipitants of *MIDN* KO HEK-293T cells co-transfected with 2xHA-GFP-Catch1 and 2xFLAG-MBP-Catch2 constructs, where “e” signifies empty 2xHA-GFP or 2xFLAG-MBP.

To gain insight into how midnolin interacts with the proteasome and its numerous substrates, we used AlphaFold to obtain a predicted structure of midnolin, which revealed three confidently predicted and highly conserved regions with defined structure (Fig. 5A, fig. S4, A and B) (26). Midnolin does not contain structural elements that are characteristic of RING- or HECT-type ubiquitin ligases (27), and does not contain a ubiquitin-binding domain characteristic of proteasomal processivity factors like Rad23 or Ubiquilin (28). Instead, midnolin contains a ubiquitin-like domain (Ubl) towards its N-terminus. Additionally, midnolin contains two discontinuous regions, each composed of two predicted anti-parallel β strands and two or three α helices, that appear to fold together to form a domain with internal symmetry. For reasons discussed below, we named this region of midnolin the “Catch” domain. Finally, midnolin contains a long α helix towards its C-terminus, termed αHelix-C, which includes a predicted nuclear localization sequence (NLS). Indeed, endogenous midnolin was largely located within the nucleus and deletion of the predicted NLS, but not other regions of midnolin, resulted in its localization to the cytoplasm (fig. S5, A and B).

To examine whether these three regions are important for midnolin function, we transiently expressed wild-type and mutant versions of midnolin in *MIDN* KO HEK-293T cells stably expressing the GPS IRF4 or FosB reporters (Fig. 5, B and C). While wild-type midnolin potently promoted IRF4 and FosB destruction, point mutations of the Ubl or deletions of the Ubl, Catch, αHelix-C, or NLS domains abrogated midnolin function (Fig. 5C, fig. S6A).

We next performed co-immunoprecipitation experiments to identify midnolin domains that are required for its interaction with substrates and/or the proteasome. Point mutations or deletion of the Ubl domain did not disrupt the stable association of midnolin with EGR1 or the proteasome (Fig. 5D). However, mutagenesis of the Ubl domain potently increased midnolin levels and these Ubl mutants were only marginally sensitive to MG132 (Fig. 5D). This suggests that the ubiquitination-independent degradation of midnolin that we observed previously (Fig. 4C) is dependent on its Ubl domain. In contrast, deletion of the NLS or entire C-terminal α helix revealed that this domain is necessary for midnolin to interact stably with the proteasome, but not its substrates (Fig. 5D). This association with the proteasome was not affected by inhibition of the proteasome or E1 ubiquitin-activating enzymes (fig. S6B). When fused to maltose binding protein (MBP), the midnolin helix conferred the ability to interact with the proteasome (Fig. 5E). Thus, the C-terminal midnolin helix is both necessary and sufficient to bind the proteasome and midnolin, unlike the processivity factors Rad23 and Ubiquilin, engages the proteasome stably independent of a Ubl domain (Fig. 5D) (29, 30).

Deletion of the regions that fold together to form the Catch domain (the N-terminal Catch1 and C-terminal Catch2 subdomains) abolished the interaction of midnolin with its substrates, without affecting its ability to bind the proteasome (Fig. 5D). In growth arrested NIH/3T3 cells where the level of endogenous midnolin substrates was induced upon serum restimulation, the interaction of midnolin with its substrates also required the Catch domain (fig. S6C). To determine if the Catch domain is both necessary and sufficient to engage midnolin substrates, we immunoprecipitated transfected wild-type, Catch domain-deleted midnolin, or the Catch domain alone in HEK-293T cells and assessed the interaction with various substrates and proteasomal components. Deletion of the Catch domain abolished the interaction of midnolin with its substrates while retaining proteasome binding, and the Catch domain alone was sufficient to bind midnolin substrates (Fig. 5F). Catch1 and Catch2 are separated by a long 111 amino acid unstructured sequence that is poorly conserved. We shortened this Catch1 and Catch2 linker to 10 amino acids (ΔLoop1), 16 amino acids (ΔLoop2), or 28 amino acids (ΔLoop3). These midnolin variants still interacted with substrates and promoted their degradation, indicating that the long stretch connecting Catch1 and Catch2 is largely dispensable (Fig. 5F, fig. S7, A and B). However, the length between Catch1 and Catch2 could not be too short, because the shortest linker (ΔLoop1, 10 amino acids) did attenuate the interaction of midnolin with certain substrates (Fig. 5F and fig. S7A). Thus, the linker between Catch1 and Catch2 may still be important for proper flexibility or folding of the Catch domain.

Given the importance of the Catch domain for binding substrates, we further validated the AlphaFold prediction that Catch1 and Catch2 fold together. This folding may be strongly driven by hydrophobicity, because the core of the Catch domain is comprised exclusively of highly conserved hydrophobic amino acids (fig. S7C). To validate that Catch1 and Catch2 fold back to bind each other in a process driven by hydrophobicity, we expressed each separately by tagging Catch1 to 2xHA-GFP and Catch2 to 2xFLAG-MBP. We also generated a series of mutants for both Catch1 and Catch2 where some hydrophobic residues that are predicted by AlphaFold to drive the interaction were changed to aspartic acid. As a control, we mutagenized a solvent-exposed residue on Catch1 (S134) and Catch2 (R301) because these were not predicted to contribute to the binding between Catch1 and Catch2. These epitope-tagged Catch1 and Catch2 constructs were expressed in *MIDN* KO HEK-293T and 2xHA-GFP-Catch1 was immunoprecipitated. Indeed, Catch1 and Catch2 bound each other when expressed as independent proteins, and mutagenesis of the hydrophobic residues to aspartic acid abolished the interaction (Fig. 5G). This contrasts with mutagenesis of the solvent-exposed residues that did not alter the Catch1-Catch2 interaction as anticipated. We also introduced these same mutations into full-length midnolin and only mutagenesis of the hydrophobic residues abolished the ability of midnolin to bind with and promote the degradation of its substrates (fig. S7, B and D). The hydrophobic core was important for the function of the Catch domain, because mutagenesis of additional hydrophobic residues to aspartic acid attenuated the ability of midnolin to promote the degradation of its substrates including IRF4 and FosB (fig. S7E).

Thus, midnolin is primarily localized within the nucleus, associates with the proteasome using its long C-terminal α helix, binds substrates using its hydrophobic Catch domain, and contains a Ubl domain that is necessary to promote substrate degradation. Overall, these three regions of midnolin function in concert to promote ubiquitination-independent proteasomal degradation of bound substrates.

## Midnolin “catches” regions within its substrates that constitute a β strand degron

**Fig. 6.**
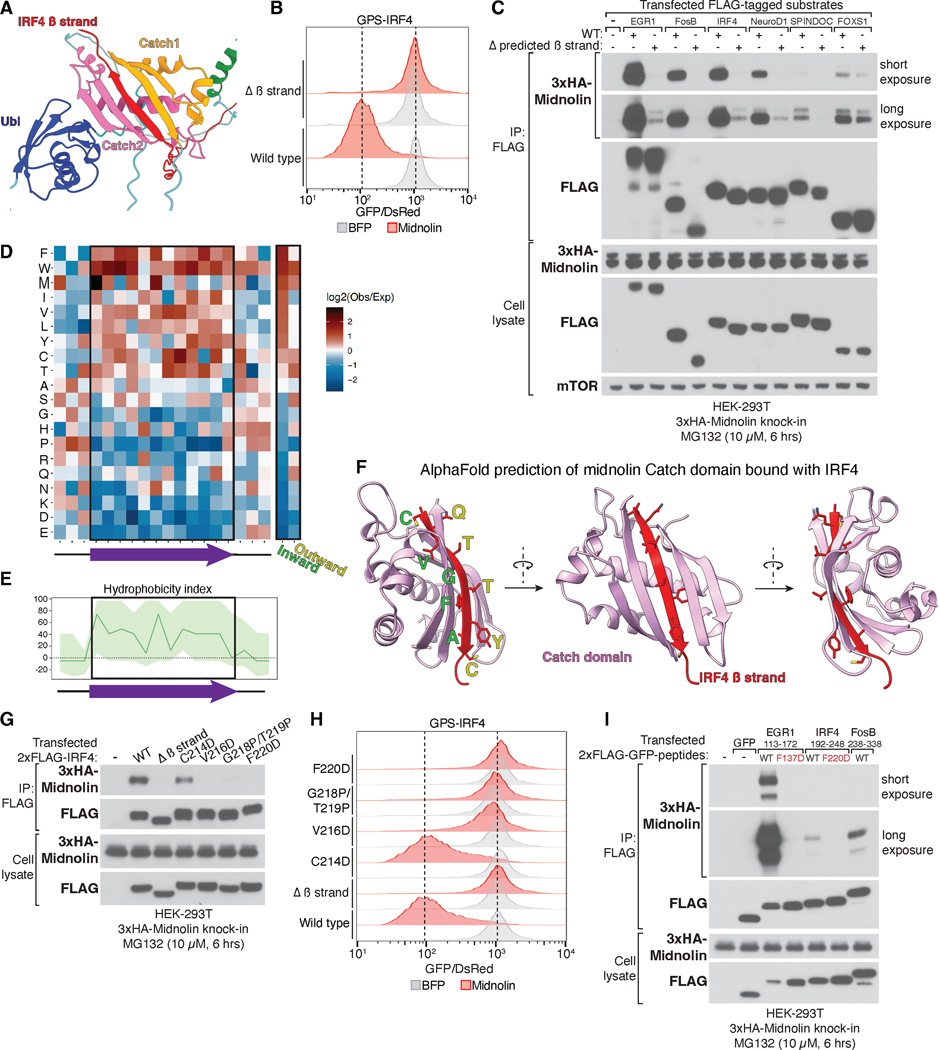
Midnolin catches regions within its substrates that constitute a β strand degron. (**A**) AlphaFold structure prediction of midnolin bound to its substrate IRF4 reveals an adopted β strand capture model. (**B**) Midnolin requires the predicted β strand within IRF4 to promote degradation. The GPS IRF4 reporters were stably expressed in *MIDN* KO HEK-293T cells and a control BFP or midnolin co-expressing BFP were transiently transfected before analyzing the GFP/DsRed ratio by flow cytometry. (**C**) Predicted β strands are necessary for interaction with midnolin. Immunoblotting was performed from anti-FLAG immunoprecipitants of 3xHA-midnolin knock-in HEK-293Ts transfected with 2xFLAG-tagged substrates. For FosB, the comparison is between the full-length protein and ΔFosB. Cells were treated with 10 μM MG132 for 6 hours. See methods for the truncation boundaries. (**D**) Amino acid frequency of midnolin substrate β strands predicted by AlphaFold reveals a strong preference for hydrophobic residues. ‘Inward’ is defined by the residues buried within the Catch domain, while ‘outward’ is defined by the solvent-exposed residues. (**E**) The hydrophobicity of residues within the β strand was determined by a mean hydrophobicity index at pH 7 (58, 59) of residues immediately preceding, within, or immediately following the β strand. (**F**) AlphaFold structure prediction of the midnolin Catch domain bound to IRF4. (**G**) Hydrophobic β strand residues buried within the Catch domain are required for midnolin interaction. Similar assay as (C) from cells transfected with the 2xFLAG-tagged IRF4 constructs. (**H**) Midnolin requires the hydrophobic β strand residues buried within the Catch domain to promote degradation. Similar assay as (B) (**I**) Regions encompassing predicted β strand(s) are sufficient for conferring an interaction with midnolin. Similar assay as (C) from cells transfected with the indicated 2xFLAG-GFP-peptide fusions.

We were curious as to how midnolin achieves substrate selection through its Catch domain given its ability to promote the destruction of many diverse proteins. Canonically, E3 ubiquitin ligases bind short linear motifs within substrates, termed degrons, in which the amino acid side chains play crucial roles in determining substrate specificity (31–34). To gain insights into how midnolin achieves substrate selection, we used AlphaFold to predict the structure of the midnolin-substrate bound complex (26). The AlphaFold predictions for midnolin bound to IRF4 (Fig. 6A) revealed that a predicted unstructured region of native IRF4 (fig. S8A) formed a β strand upon binding to the midnolin Catch domain (fig. S8B), thereby completing a five-stranded anti-parallel β sheet tertiary structure. Consistent with this AlphaFold predicted interaction, a small deletion within IRF4 that encompassed the predicted β strand abolished the ability of midnolin to promote IRF4 degradation (Fig. 6B).

To determine the generality of this β strand capture mechanism, we performed the AlphaFold structure predictions for the 508 most destabilized proteins uncovered in the GPS ORFeome screen. The PDB files for these 508 predictions can be accessed using the following DOI (10.5061/dryad.m905qfv6g). Approximately 40% (205/508) of the proteins have predictions that are consistent with them being captured by midnolin (Data S4). We systematically compared the AlphaFold-predicted structure of midnolin substrates in the native and midnolin-bound states. The regions predicted to be captured by midnolin are generally more unstructured than the rest of the same protein in the native state but have the potential to form a β strand upon binding the Catch domain (fig. S8C). We validated these findings by introducing into several representative midnolin substrates small deletions that encompassed the predicted β strand and tested the ability of midnolin to interact with and promote the degradation of these mutants. In each case, deletion of the predicted β strand abrogated the ability of midnolin to both interact with substrates (Fig. 6C) and promote their degradation (fig. S9, A to D).

The AlphaFold predictions yielded several different modes of midnolin-FosB interaction, raising the possibility that the N- and C-terminus of FosB have the potential to form multiple different β strands that could be captured by midnolin. We generated various truncated forms of FosB to determine which regions were necessary for midnolin to promote degradation and found that the C-terminal 101 amino acids were required (fig. S10A). Truncation of the last 101 amino acids of FosB results in a naturally existing splice isoform termed ΔFosB, which was shown to be significantly more stable than other Fos family members, although the molecular basis for this increased stability was unknown (35). Previous studies have shown that chronic and repeated exposure to drugs of abuse such as cocaine leads to week-long accumulation of ΔFosB protein expression in the *nucleus accumbens*, a brain region crucial for addiction (35, 36). Indeed, overexpression of ΔFosB in neurons of the *nucleus accumbens* sensitizes animals to the effects of cocaine and may thus contribute to addiction (37). In contrast to full-length FosB, midnolin weakly interacted with and less effectively promoted the degradation of ΔFosB (Fig. 6C and fig. S10A). Thus, our findings provide a mechanistic explanation for the increased stability of ΔFosB. Nevertheless, ΔFosB is eventually degraded, and this may require the N-terminus of FosB, which is also predicted by AlphaFold to form a β strand that can be captured by midnolin. Deletion of the predicted N-terminal β strand region within ΔFosB largely abolished its ability to be targeted for decay by midnolin (fig. S10B). Thus, a protein can contain more than one region that can be captured by midnolin.

Having identified sequences that mediate the midnolin-substrate interaction, we asked whether specific amino acids were enriched or depleted over others within the predicted β strands captured by the midnolin Catch domain. Within these β strands, we observed a marked depletion of charged amino acids including aspartic acid and glutamic acid, as well as proline (Fig. 6D), which is known to disrupt β strands (38). Instead, there was a strong enrichment for hydrophobic amino acids within the midnolin induced β strands, which overall are significantly more hydrophobic than the average of all other regions within the same protein (Fig. 6E and fig. S10C). Once bound by midnolin, these hydrophobic β strand residues were predicted to be buried within the core of the Catch domain (inward), while charged amino acids tended to point outward and were solvent exposed (Fig. 6, D and F, and fig. S10D). The enrichment for hydrophobicity within the β strands is consistent with the fact that the core of the Catch domain is also highly hydrophobic and required for catching hydrophobic regions within substrates. To validate these predictions, two residues (G218 and T219) at the center of the predicted IRF4 β strand were mutagenized to proline to potentially disrupt β strand formation, or the hydrophobic residues buried within the interior of the Catch domain (V216, and F220) were mutagenized to aspartic acid to disrupt potential hydrophobic interactions. Consistent with the AlphaFold predictions, both introduction of prolines and mutagenesis of the hydrophobic residues to aspartic acid within the β strand abolished the ability of midnolin to interact with (Fig. 6G) and promote the degradation of IRF4 (Fig. 6H). Similar results were obtained following mutagenesis of the corresponding residues within the EGR1 adopted β strand (fig. S10, E and F).

We next tested whether midnolin can interact with and promote the degradation of GFP fused to a short sequence containing the β strand degron(s) within substrates. Midnolin interacted with (Fig. 6I) and promoted the degradation of (fig. S10, G and H) GFP fused to short stretches within EGR1, IRF4, and the C-terminal tail of FosB that was dependent on the hydrophobicity of the predicted β strand. Thus, the β strands predicted by AlphaFold are necessary and sufficient to interact with midnolin for proteasomal degradation.

We conclude that midnolin achieves substrate selection via a general mechanism: captured regions within substrates possess the ability to form a β strand that is biochemically compatible with the hydrophobic core of the Catch domain.

## Discussion

In this study, we identified a protein called midnolin that targets stimulus-induced transcription factors, such as c-Fos, FosB, EGR1, NR4A1, and IRF4, to the proteasome for degradation. Disrupting midnolin function in various cell types increases the peak abundance of these transcription factors and prolongs their expression. It is not uncommon for a protein to be targeted for proteasomal degradation by multiple mechanisms and these stimulus-responsive transcription factors may not be targeted for degradation solely by midnolin. Rather, it is possible that these proteins are also targeted by a ubiquitination-dependent pathway that functions in parallel with midnolin and could be the primary mechanism of degradation in some contexts. Indeed, it has been reported that Fos family members are targeted by both ubiquitination-dependent and -independent mechanisms (7). We found that midnolin is induced by diverse stimuli, and its induction may act as a post-translational feedback circuit to limit the time course of expression of these stimulus-responsive transcription factors. Through a gain-of-function genetic screen, we identified a large group of potential targets of midnolin that is strongly enriched for nuclear proteins, especially transcriptional regulators, revealing that midnolin functions broadly to promote the degradation of proteins in the nucleus where midnolin itself is predominantly localized.

The biological functions of midnolin are likely complex. Many of its substrates play central roles in the nervous and immune systems, and thus it will be important to establish the physiological function of midnolin in vivo. Midnolin was initially discovered due to its strong induction in the midbrain early during embryonic development (39). How midnolin expression is induced both during development and in cultured cells is currently unclear. Midnolin may have evolved to integrate various upstream stimuli and shape the proteome swiftly as a crucial response to the initial stimuli. Many midnolin substrates including IEG proteins and lineage-specific transcription factors undergo a transient burst of expression either in response to a particular stimulus or during a specific stage of development, and thus the prolonged expression of these transcription factors could be detrimental to organismal physiology. Indeed, previous studies have shown that chronic and repeated exposure to drugs of abuse such as cocaine leads to week-long accumulation of ΔFosB, which plays a crucial role for addiction in the *nucleus accumbens* of the brain (35–37). Our finding that ΔFosB is resistant to midnolin-dependent degradation provides a glimpse into the role that midnolin could play in brain function. Given that many IEG proteins are efficiently targeted for degradation by midnolin and that the precise expression of IEGs is critical for learning and memory (1, 3, 40), it is possible that disrupting or boosting midnolin function could impact the ability of animals to efficiently learn and store information in the brain.

IRF4, another midnolin substrate, is a lineage-specific transcription factor that is essential for the function and homeostasis of mature B and T cells and is an oncogenic driver of multiple myeloma (15, 16, 41). IRF4 protein expression is potently induced by diverse immunological stimuli including T cell receptor signaling, cytokines, and in fasting adipocytes (42–45). How the IRF4 protein returns to baseline after stimulation was unknown, and our finding that IRF4 is potently targeted for degradation by midnolin may provide insights into the function of midnolin in the immune system. Taken together, these findings suggest that midnolin may serve as a key regulator that determines the precise expression kinetics of stimulus-induced transcription factors by controlling their protein stability in various cell types or tissues. In principle, midnolin could be used to target different proteins under different circumstances. For example, in flies, it was reported that a midnolin ortholog, Stuxnet, is cell cycle regulated and promotes Pc protein degradation in mitosis during development (14).

**Fig. 7.**
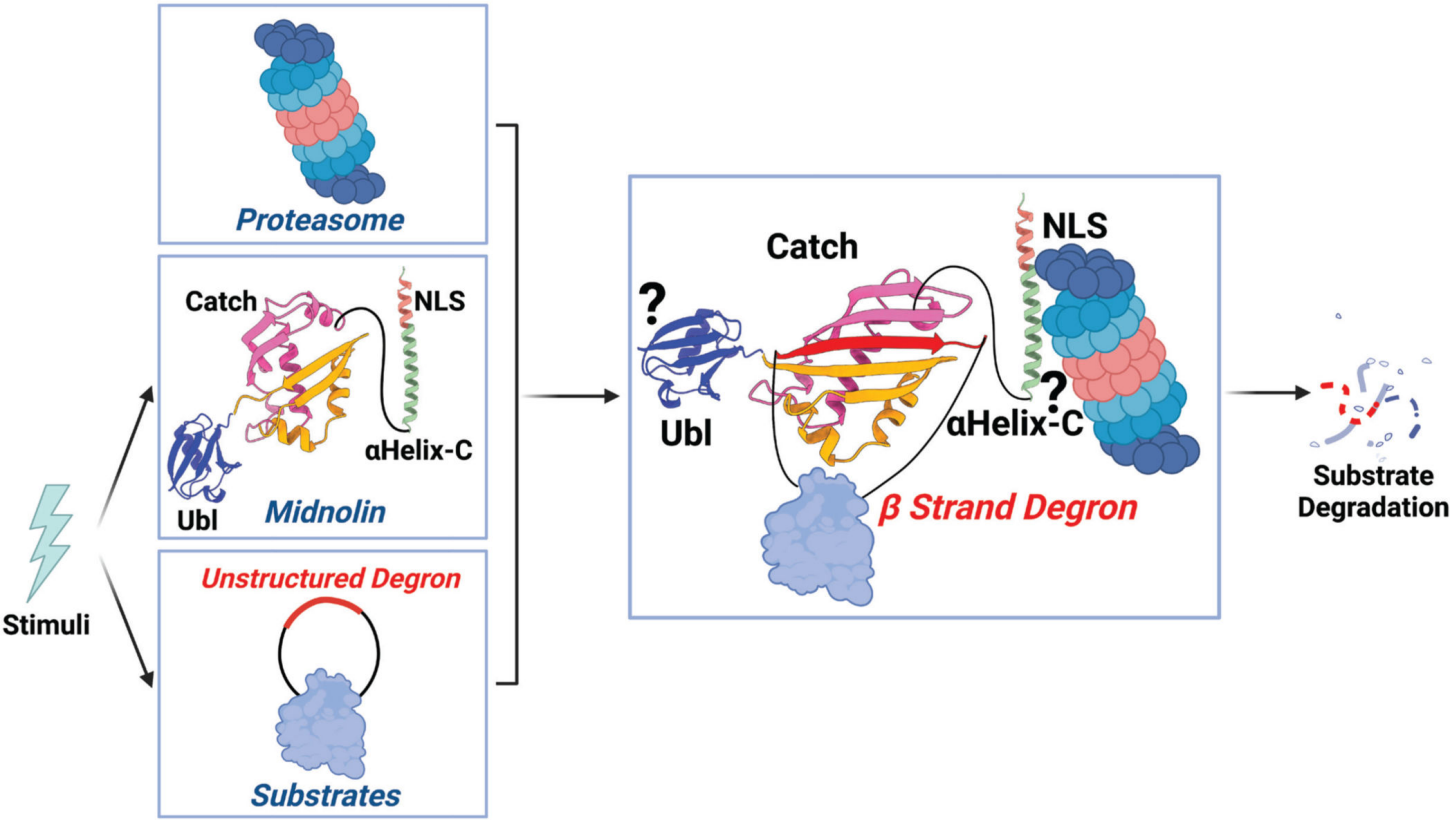
Model for how midnolin functions to promote ubiquitination-independent proteasomal degradation. Midnolin is induced by growth factors and neurological stimuli and its overexpression is sufficient to cause the degradation of its targets including transcription factors such as c-Fos, FosB, ERG1, NR4A1, IRF4, and potentially many other proteins within the nucleus, where midnolin primarily resides. The degradation of its substrates does not require ubiquitination. Instead, midnolin utilizes its Catch domain to bind unstructured hydrophobic regions within substrates that have the potential to form a β strand that functions as a midnolin degron. Midnolin associates with the proteasome using its long C-terminal α helix and promotes the destruction of Catch-bound substrates via its N-terminal ubiquitin-like domain. Structures of the midnolin domains are derived from AlphaFold predictions. How the C-terminal α helix of midnolin binds the proteasome, whether a conformational change occurs after substrate binding, and how the ubiquitin-like domain confers degradative activity require further investigation (created with BioRender.com).

We found that midnolin promotes the degradation of its targets in a proteasome-dependent, but ubiquitination-independent manner. This is supported by multiple lines of evidence. Removal of lysine residues from many midnolin targets did not abrogate the ability of midnolin to bind with and promote their destruction. A caveat to this interpretation is that ubiquitination can occur on the N-terminal amine of the first amino acid if it is not acetylated, or on other residues such as cysteine, serine, or threonine more rarely (24). However, in contrast to the effects of proteasomal inhibition, blocking the E1 ubiquitin-activating enzyme did not abrogate midnolin function while stabilizing canonical substrates of the ubiquitin-proteasome system. Together, these experiments allowed us to conclude that midnolin does not require ubiquitination for its degradative function. Instead, midnolin engages substrates using its Catch domain, which binds a hydrophobic region capable of β strand formation that functions as a degron. Midnolin associates with the proteasome using its long, C-terminal α helix and contains an N-terminal ubiquitin-like domain that is essential to promote the degradation of bound substrates. We hypothesize a model whereby the concerted action of these three regions of midnolin allows for the ubiquitination-independent proteasomal degradation of midnolin-bound proteins (Fig. 7).

Previous work demonstrated that ornithine decarboxylase (ODC) is targeted directly to the proteasome for degradation via a C-terminal unstructured sequence (46–49). However, the regions within substrates captured by midnolin are unlike any others previously described for E3 ubiquitin ligases. The midnolin degron appears to be generally unstructured, but has potential to form a β strand upon binding midnolin, with aliphatic residues of the degron buried within the hydrophobic core of the Catch domain. The integration of the β strand degron into the Catch domain appears to complete a five-stranded anti-parallel β sheet structure. The somewhat degenerate nature of these hydrophobic degrons in various substrate proteins is likely explained by the fact that β sheets are stabilized not only by side chain interactions, but also by backbone hydrogen bonding (50), which may reduce the need for specific amino acid interactions. A region with the propensity to form a β strand that is also biochemically compatible with the hydrophobic core of the Catch domain may be present in many proteins, thereby explaining how midnolin recognizes a diverse set of proteins. Given the ubiquity of such β strands as structural elements of proteins, midnolin recognition of unfolded proteins could behave as a general quality control mechanism.

How midnolin initiates the degradation of bound substrates is not completely understood mechanistically. We do not know if midnolin interacts with its targets before binding to the proteasome, or whether midnolin associates with the proteasome constitutively and then recruits its substrates, thus defining a new subclass of proteosomes in the nucleus. We favor the latter possibility, as a unique feature of midnolin is that it interacts stably with the proteasome using a C-terminal α helix but not its Ubl domain. This is unlike the processivity factors Rad23 and Ubiquilin that bind ubiquitinated cargo and utilize their Ubl domain to interact with the proteasome (29, 30, 51–53). How the midnolin α helix binds the proteasome is currently unclear and we do not yet understand how the Ubl functions. However, the Ubl domain of midnolin is necessary for the degradation of bound substrates and it is noteworthy that midnolin itself is efficiently degraded by the proteasome in a ubiquitination-independent manner that requires the Ubl domain. Whether midnolin is reused or being degraded along with the substrate remains to be determined. We hypothesize that when midnolin interacts with a substrate, a conformational change occurs that allows the proteasome to recognize the Ubl domain transiently to promote the degradation of the bound substrate, with or without midnolin also being degraded. Structural and biochemical analyses are required to deepen our understanding of this non-canonical docking and degradative mechanism.

Our results suggest the midnolin-proteasome pathway may represent a general mechanism by which the proteasome bypasses the traditional ubiquitination system to achieve selective degradation of many nuclear proteins. It has been reported that in bacteria, which do not contain the ubiquitin-proteasome system, a hierarchy of adaptors mediate selective degradation of diverse proteins by the proteasome-equivalent ClpXP protease complex (54, 55). Thus, it will be of interest to determine whether, in the course of evolution, additional proteins have evolved to recruit proteins directly to the proteosome for degradation.

## Materials and Methods

### Cell culture

HEK-293T (ATCC, CRL-3216, RRID: CVCL_0063) and NIH/3T3 cells (ATCC, CRL-1658, RRID: CVCL_0594) cells were cultured at 37°C and 5% CO_2_ in Dulbecco’s Modified Eagle’s Medium (DMEM) (Thermo Fisher Scientific, 11965118) supplemented with 100 units/mL of penicillin and 0.1 mg/mL of streptomycin (Thermo Fisher Scientific, 15070063) and 10% fetal bovine serum (Cytiva, SH30088.03). Ramos B cells (ATCC, CRL-1596, RRID: CVCL_0597) were cultured in RPMI 1640 medium (Thermo Fisher Scientific, A1049101) supplemented with 100 units/mL of penicillin and 0.1 mg/mL of streptomycin and 10% fetal bovine serum. NIH/3T3 cells were starved overnight of serum and restimulated the following day with 20% serum for the indicated time points.

Animals were handled according to the protocol (IS00000074–6) approved by the Harvard University Office of the Institutional Animal Care and Use Committee, HMA Standing Committee on Animals and were in accordance with federal guidelines. Mouse cortical neurons were isolated and cultured as described previously (61). In short, embryonic cortices from wild-type C57BL/6NCrl mice (Charles River Laboratories, strain number: 027; 5–10 embryos: both males and females) were dissected at E16.5, and dissociated with papain (Sigma Aldrich, 10108014001). After terminating the papain digestion with ovomucoid (trypsin inhibitor from Worthington), cells were triturated gently through a P1000 pipette before passing through a 40-micron filter, then plated on cell culture dishes coated with poly-D-lysine (20 μg/mL) and laminin (4 μg/mL). The culture medium used for neurons was Neurobasal medium (GIBCO) that contains 2% B27 supplement, penicillin-streptomycin (50 U/mL penicillin and 50 U/mL streptomycin), and glutaMAX (1 mM). The neurons were cultured at 37°C and 5% CO_2_, treated with corresponding viruses at 3 days in vitro (DIV) while adding fresh culture medium at the same time (35% of total volume), silenced on 10 DIV overnight by the addition of 1 μM TTX (Abcam ab120055) and 100 μM AP5 (Thermo Fisher 01–061-0), and harvested at 11 DIV after treatment with KCl stimulation buffer comprised of: 52.8 mM KCl, 0.62 mM CaCl_2_, 0.31 mM MgCl_2_, 3.1 mM HEPES pH 7.4 for the indicated times.

Cells were treated with 20 ng/mL phorbol 12-myristate 13-acetate (PMA) (Thermo Fisher Scientific, J-63916-MA), 10 μM MG132 (Selleckchem, S2619), or 500 nM TAK-243 (Selleckchem, S8341) from 1000x stock solution in DMSO for 6 hours unless stated otherwise.

### Plasmids and Cloning

The genome-wide CRISPR-Cas9 sgRNA Root library (5 sgRNAs/gene, 94,335 sgRNAs total) was used previously and the sgRNA information can be found in Data S1 (62). The barcoded GPS ORFeome expression library was generated previously (13). The plasmids for human cDNAs containing a stop codon and conferring kanamycin resistance were obtained from the Ultimate ORF Clone collection (Thermo Fisher Scientific) in the form of Gateway entry clones: MIDN (IOH62653, BC094778.1), FOSB (IOH62162, NM_006732.2), FOS (IOH5624, NM_005252.3), ATF2 (IOH37849, NM_001880.2), CREB3 (IOH14437, BC009402.2), CREB5 (IOH53714, NM_001011666.1), ATF3 (IOH6465, NM_001674.2), BATF2 (IOH13295, BC012330.1), COMMD9 (IOH12792, NM_014186.3), C11ORF31 (IOH58679, NM_170746.2), ZNF621 (IOH44483, NM_198484.3), SERTAD2 (IOH42292, NM_014755.2), LMX1A (IOH34878, NM_177398.2), LMX1B (IOH34707, NM_002316.2), HOXD3 (IOH5660, NM_006898.4), CHCHD2 (IOH3869, NM_016139.2), FNDC3B (IOH10620, BC012204.1), SOX12 (IOH40697, NM_006943.2), TAX1BP3 (IOH13074, NM_014604.2), PAX4 (IOH34754, BC074761.2), FOXA3 (IOH10014, NM_004497.2), STYX (IOH10157, NM_145251.3), ZNF764 (IOH6451, NM_033410.2), RelB (IOH11686, NM_006509.2), PRRX1 (IOH36664, NM_006902.3), IRF1 (IOH2022, NM_002198.2), IRF2 (IOH10126, NM_002199.3), IRF8 (IOH42114, NM_002163.2), IRF9 (IOH28745, NM_006084.4), MYC (IOH2954, P01106), SPINDOC (IOH28799, NM_138471.1), PPDPF (IOH4080, NM_024299.2), PAX8 (IOH3823, NM_003466.3), FOXS1 (IOH13387, NM_004118.3), NEUROD1 (IOH3394, NM_002500.2), MIER2 (IOH40210, NM_017550.1), IRF4 (IOH12141, NM_002460.2), GATA1 (IOH57792, NM_002049.3), CITED1 (IOH5542, BC004240.1), XRCC1 (IOH40644, NM_006297.2).

The plasmids for human cDNAs lacking a stop codon and conferring spectinomycin resistance were obtained from the Human ORFeome library V8.1 (Dana Farber Cancer Institute) in the form of Gateway entry clones: EGR1 (ORF_ID #14665, BC073983.1) and NR4A1 (ORF_ID #292, BC016147.1).

CBX4 and HA-tagged WT or K to R cDNA were generated by synthesis using IDT with attB1 and attB2 overhangs for cloning into the pDONR221 (Thermo Fisher Scientific, 12536017) via a BP recombination reaction (Thermo Fisher Scientific, 11789020) to generate the entry clone. Similarly, the following attB1 and attB2 overhangs we included in primers to generate fragments for sufficiency experiments by PCR for cloning into the pDONR221:

attB1 - GGGGACAAGTTTGTACAAAAAAGCAGGCTTAgccacc

attB2 - GGGGACCACTTTGTACAAGAAAGCTGGGTA

Entry clones were mutagenized by PCR using the Q5 Site Directed Mutagenesis kit (NEB, E0554S) and the primers for mutagenesis were designed using the NEBaseChanger program.

Midnolin amino acid sequence used:

MEPQPGGARSCRRGAPGGACELGPAAEAAPMSLAIHSTTGTRYDLAVPPDETVEGLRKRLSQRLKVPKERLALLHKDTRLSSGKLQEFGVGDGSKLTLVPTVEAGLMSQASRPEQSVMQALESLTETQVSDFLSGRSPLTLALRVGDHMMFVQLQLAAQHAPLQHRHVLAAAAAAAAARGDPSIASPVSSPCRPVSSAARVPPVPTSPSPASPSPITAGSFRSHAASTTCPEQMDCSPTASSSASPGASTTSTPGASPAPRSRKPGAVIESFVNHAPGVFSGTFSGTLHPNCQDSSGRPRRDIGTILQILNDLLSATRHYQGMPPSLAQLRCHAQCSPASPAPDLAPRTTSCEKLTAAPSASLLQGQSQIRMCKPPGDRLRQTENRATRCKVERLQLLLQQKRLRRKARRDARGPYHWSPSRKAGRSDSSSSGGGGSPSEASGLGLDFEDSVWKPEANPDIKSEFVVA

Midnolin regions for truncations or sufficiency experiments:

ΔUbl - Residues 31–105

ΔCatch1 - Residues 112–156

ΔCatch2 - Residues 266–332

ΔNLS - Residues 402–413

ΔαHelix-C - Residues 377–413

αHelix-C region fused to MBP – Residues 360–432

Catch domain alone for sufficiency experiment - Residues 102–334

ΔLoop1 – Residues 160–260

ΔLoop2 – Residues 163–257

ΔLoop3 – Residues 169–251

Catch1 fused to 2xHA-GFP – Residues 107–166

Catch2 fused to 2xFLAG-MBP – Residues 254–337

FosB truncations:

Deletion1 - Residues 2–42

Deletion2 - Residues 43–82

Deletion3 - Residues 83–122

Deletion4 - Residues 123–149

Deletion5 - Residues 150–237

ΔFosB - Residues 238–338

Predicted β strand truncations:

FOXS1 - Residues 245–260

CBX4 - Residues 538–558

NEUROD1 - Residues 277–288

SPINDOC - Residues 314–328

IRF4 - Residues 208–229

FOSB (N-terminal β strand) - Residues 67–75

EGR1 - Residues 128–145

β strand sufficiency peptides:

EGR1 - Residues 113–172

IRF4 - Residues 192–248

FosB - Residues 238–338

Entry clones were subcloned into the following lentiviral Gateway destination vectors using an LR recombination reaction (Thermo Fisher Scientific, 11791100): pHAGE-GPS 3.0 (13), pHAGE-GPS 3.2 (63), pHAGE-CMV-2xFLAG-N (this paper), pHAGE-CMV-2xHA-N (this paper), pHAGE-CMV Puromycin (64), pHAGE EF1α BFP (this paper), CMV-C-2xFlag expression vector (Addgene, 118372), pHAGE TRE Blasticidin (this paper), pHAGE-CMV-2xHA-GFP-N (this paper), pHAGE-CMV-2xFLAG-GFP-N (this paper), and pHAGE-CMV-2xFLAG-MBP-N (this paper). The pInducer20 mouseCD19 (64) plasmid was used to generate dox-on responsive HEK-293T cells.

Lentiviral CRISPR-Cas9 vectors containing the sgRNA of interest were cloned by first digesting the backbone lentiCRISPRv2 (Addgene, 52961) using BsmBI (NEB, R0739S). Then, sgRNA oligos containing CACC or AAAC overhangs were obtained from IDT, phosphorylated and annealed together, and ligated into the linear backbone by T4 (NEB, M0202S) ligation.

Non-targeting sgControl - GTATTACTGATATTGGTGGG

Human sgMIDN #1 - GAAGCTGCAGGAGTTCGGCG

Human sgMIDN #2 - GCTGACCTTGGTACCCACCG

Mouse sgMIDN - GCGAGCTGAACACGGCCA

### Lentivirus production

Lentivirus was generated by transfecting HEK-293T cells using PolyJet (SignaGen, SL100688) following the manufacturer’s instructions with plasmids encoding Tat, Rev, Gag-Pol, and VSV-G and lentiviral transfer vectors. Specifically, plasmid DNA was diluted into DMEM lacking supplements, and 3 μL of PolyJet reagent was used per 1 μg of plasmid DNA. One day post-transfection, the media was removed, and the cells were supplemented with fresh complete culture media. The lentiviral supernatant was collected 48- and 72-hours post-transfection, passed through a 0.45 μm filter, and either applied directly to cells or stored at −80°C for later use. For most experiments, lentivirus was packaged in 6-well plates. For library preparations, lentivirus was packaged in 8 15 cm plates using 13 μg of total DNA per plate for transfection, pooled, concentrated using the lenti-X concentrator (Takara, 631232), and aliquoted.

### Midnolin Overexpression and Flow cytometry

Cells were seeded in 6-well plates at 200,000 cells/well and were transfected two days later using Polyjet with EF1α Midnolin co-expressing BFP, or BFP alone as a negative control. The media was changed one day post-transfection and cells were analyzed two days post-transfection.

Cells were prepared for flow cytometry by aspirating old media and rinsing cells once with PBS. The cells were detached using 0.05% trypsin at room temperature and the trypsin was neutralized using fresh complete media. The cells were then analyzed on a CytoFLEX S flow cytometer (Beckman Coulter, V2-B2-Y3-R2 version #C09762) and the CytExpert software (Beckman Coulter) was used to collect flow cytometry data. All flow cytometry data was analyzed using the FlowJo software. For fluorescence-activated cell sorting (FACS), a Sony MA900 was used for routine sorting of single clones, while a MoFlo Astrios (Beckman Coulter) instrument was used to collect cells for the CRISPR-Cas9 and GPS ORFeome screens.

### Generating doxycycline-inducible HEK-293T cells

Wild-type and *MIDN* KO HEK-293T cells were infected with lentivirus encoding the pInducer20 system expressing mouse CD19 as a doxycycline-inducible marker for cell-surface staining. After infection, the cells were treated with doxycycline (100 ng/mL) for 2 days and were stained on the cell surface using PE anti-mouse CD19 antibody (Biolegend, 152407, RRID: AB_2629816) for 30 minutes using 1 μL of antibody diluted in 100 μL of media containing 1 million cells. The cells were rinsed twice with PBS before sorting for the PE-positive population. The sorted cells were allowed to expand for more than 1 week before restaining the cells using PE anti-mouse CD19 antibody in the absence of doxycycline induction. The PE-negative population was sorted, and the cells were expanded before another round of staining and sorting for the PE-negative population. This allowed for a population of cells that responded to doxycycline with minimal leakiness. Finally, lentivirus encoding the pHAGE TRE-Midnolin was infected into these *MIDN* KO HEK-293T cells and selected with blasticidin to allow for stable, doxycycline-inducible expression of midnolin.

### EGR1 and FosB CRISPR-Cas9 screens

Genome-wide CRISPR-Cas9 screens were performed to uncover regulators of EGR1 and FosB protein stability. Specifically, the plasmid library was packaged into lentivirus by transfecting HEK-293T cells using PolyJet as described earlier, and the lentivirus was titered to obtain a multiplicity of infection around 0.3. HEK-293T cells were generated to express the GPS 3.0 FosB or GPS 3.2 EGR1 reporters by selecting using hygromycin (200 μg/mL). These cells were then transduced with the titered CRISPR-Cas9 genome-wide Root library lentivirus at an MOI ~0.3 to maintain a 500x representation throughout. Cells were selected 48 hours post-transduction for 7 days using puromycin (2 μg/mL) to remove uninfected cells. On the ninth day of puromycin selection, the 95^th^ percentile most stable cell population was collected based on the GFP/DsRed ratio by FACS using a MoFlo Astrios instrument (Beckman Coulter). Additionally, the unsorted input cells were collected based on the number of cells collected in the enriched population. Collected cells were rinsed once with PBS, pelleted, and stored in −80°C.

### Midnolin GPS ORFeome screen

The GPS ORFeome screen was performed as described previously (13) with some modifications. Sufficient cell numbers were used to maintain at least a 300-fold coverage of the library throughout. The library was packaged into lentivirus, which were used to transduce *MIDN* KO HEK-293T at a multiplicity of infection of 0.2. Two days post-transduction, the HEK-293T cells were treated with 2 μg/mL of puromycin for 6 days to remove uninfected cells, passaging the library once in between the selection period. The library-expressing cells were plated at 4 million cells/plate in a 15 cm dish and were transfected two days later using Polyjet with 8 μg DNA of EF1α-Midnolin co-expressing BFP, or BFP alone as a negative control. The cells were harvested two days post-transfection and were sorted into six stability bins based on the GFP/DsRed ratio by FACS using a MoFlo Astrios instrument (Beckman Coulter). The sorting gates were established using the BFP control to ensure 1/6^th^ of the population was collected per bin. Once the control populations were collected, the cells overexpressing midnolin were partitioned using the exact same sorting and gating settings as the control. The collected cells from each stability bin were rinsed once with PBS, pelleted, and frozen at −80°C for at least 12 hours.

### Deconvolution of the pooled screens

Cell pellets were thawed, and genomic DNA was harvested using a Gentra Puregene Core Kit, Qiagen. The sgRNAs or barcodes were then amplified by PCR using all the genomic DNA as template (4 μg DNA per reaction) to include stagger sequences and Q5 Hot Start High-Fidelity DNA Polymerase from NEB. A second round of PCR was performed using the clean PCR1 product to add the Illumina P5 and P7 adaptor sequences. PCR2 samples were cleaned, pooled in the correct ratio, and sequenced on a NextSeq 500 instrument. The abundance of sgRNAs or barcodes were extracted from the raw sequencing data using Cutadapt (65) and were mapped onto the reference library using Bowtie2 (66).

MAGeCK was used to determine the enrichment of sgRNAs in the 95^th^ percentile relative to the input population (56). The MAGeCK score plotted on the Y-axis represents the negative log10 of the “pos|score” value generated by MAGeCK.

For the GPS ORFeome analysis, the abundance of each ORF was corrected to account for sequencing depth and a protein stability index (PSI) score between 1 (most unstable) and 6 (most stable) was calculated using the following formula for each extracted ORF: $PSI=\sum_{i=1}^{6}Ri*i$, where *i*=the number of the stability bin denoted as an integer and R*i* = the Illumina read proportion extracted from the bin *i*. The change in protein stability between midnolin and BFP is denoted as the difference in PSI (ΔPSI).

### Gene set enrichment of GPS ORFeome hits

Gene set enrichment analyses (GSEA) was performed with GSEAPreranked (67) (v4.3.2) using rank weights derived from GPS ORFeome ΔPSI values as input. These were tested for enrichment across the Human Molecular Signatures Database (68) (MSigDB v2022.1.Hs) C5 Gene Ontology collection (i.e. GO:BP, GO:MF, and GO:CC). The classic Kolmorogorov-Smirnov scoring scheme was used with 10,000 permutations and excluded gene sets with <10 or >1000 entries when intersected with the list of GPS ORFs.

### Generating *MIDN* knockout cells

To generate isogenic single clones lacking midnolin, HEK-293T cells were transfected with the lentiCRISPRv2 BFP plasmid encoding the given sgRNA of interest using Polyjet. Several days post-transfection, the BFP-positive cells were collected as single cells into 96-well plates using FACS. The single clones were allowed to expand for 2 weeks before screening for a knockout phenotype by immunoblotting as well as next generation sequencing of the genomic DNA locus encompassing the cut-site (fig. S11A).

To generate a population-level depletion of midnolin in mouse NIH/3T3 fibroblasts, cells were transduced with lentivirus encoding the given mouse lentiCRISPR v2 sgRNA co-expressing puromycin. After 3 days post transduction, cells were selected by 2 μg/mL puromycin for 5 days and expanded for further analysis.

To generate a population-level depletion of midnolin in primary cortical neurons, cells were transduced with lentivirus encoding the given mouse lentiCRISPRv2 sgRNA co-expressing puromycin.

To generate a population-level depletion of midnolin in Ramos B cells, the cells were transduced by spinfection with lentivirus encoding the given lentiCRISPRv2 sgRNA#1 co-expressing BFP. Specifically, the cells were incubated with lentivirus for 30 minutes with centrifugation at 2000 rpm at room temperature. The cells were allowed to expand for 6 days post transduction and the BFP-positive cells were collected by FACS.

### Generating 3xHA-MIDN knock-in HEK-293T cells

To generate an endogenous 3xHA-tagged midnolin cell line, we employed the power of homology-directed repair (HDR) and CRISPR-Cas9. We reasoned that an N-terminal epitope tag would be tolerable, as an N-terminal tagged midnolin transgene could interact with substrates and promote their degradation. To establish the knock-in line, the following custom Alt-R gRNA and ssDNA HDR template were generated by synthesis from IDT:

#### gRNA:

rCrCrGrGrGrCrUrGrCrGrGrCrUrCrCrArUrCrCrCrGrUrUrUrArArGrArGrCrUrArUrGrCrUrGrGrArArArCrArGrCrArUrArGrCrArArGrUrUrUrArArArUrArArGrGrCrUrArGrUrCrCrGrUrUrArUrCrArArCrUrUrGrArArArArArGrUrGrGrCrArCrCrGrArGrUrCrGrGrUrGrCrUrUrUrUrUrUrU

#### ssDNA Template:

CGGCGCCCGCCGCCCCCAGCCCCCCAGCGCGCGCCGGGGATGTATCCCTATGACGTGCCTGATTACGCCGGCGGAGGATCCTACCCCTATGATGTGCCTGACTACGCTGGCAGCGGAGGATACCCTTATGATGTGCCTGATTATGCTGGAGGTGGAGGTAGTGAGCCGCAGCCCGGCGGCGCCCGGAGCTGCCGGCGCGG

spCas9, gRNA, and ssDNA template were introduced to cells by nucleofection. Specifically, per nucleofection of 100,000 cells in Lonza strip nucleofector system (V4XC-2032), mix 0.8 μL of 62.1 μM spCas9 (Aldevron, 9212–0.25MG), 0.8 μL of 100 μM gRNA (Alt-R from IDT), 0.25 μL of 10x NEB Buffer 3.1 (B6003S), and 0.65 μL of H_2_O to bring the final volume to 2.5 μL per nucleofection and incubate RNP at room temperature for 30 minutes. Making a master mix to troubleshoot the ssDNA template concentration is recommended to avoid small volumes. Then, add 2 μL of the RNP to 100,000 HEK-293T cells resuspended in 16.4 μL of Nucleofector™ solution plus 3.6 μL Supplement. Donor DNA was added directly to this solution at a final concentration of 500 nM or 2 μM. Cells were nucleofected using a 4D Nucleofector™ X Unit and a GFP positive control was included to ensure nucleofection worked properly. Once nucleofected, the cells were allowed to recover for 10 minutes at room temperature before adding the cells to 6-well plates containing warm media. The cells were allowed to expand for several days, and a cell lysate was collected to ensure the endogenous editing worked by immunoblotting. Then, single cells were partitioned into 96-well plates to obtain isogenic clones, which were validated by immunoblotting once expanded to ensure successful HA knock-in.

### Immunoprecipitation

Cells stably expressing the indicated epitope-tagged protein were cultured in 10 cm or 15 cm plates and allowed to reach 90% confluency before lysis. Alternatively, cells in 10 cm dishes were transiently transfected with 3 μg of the indicated plasmid DNA using Polyjet when 50% confluent. The media was changed one day post-transfection and the cells were lysed two days post-transfection with or without any necessary perturbations, such as proteasomal inhibition. For lysis, cells were rinsed once with ice-cold PBS by pouring and collected by scraping in 0.7 mL (10 cm plate) or 1 mL (15 cm plate) of lysis buffer containing 0.5% CHAPS, 40 mM HEPES pH 7.4, 100 mM NaCl, 4 mM EDTA, supplemented with 1x protease and phosphatase inhibitor cocktail (Thermo Fisher Scientific, 78441). Cell lysates were incubated with end-to-end rotation at 4°C for 30 minutes before clarification by centrifugation at 21,000xg for 15 minutes at 4°C. Anti-FLAG (Sigma, M8823, RRID: AB_2637089) or anti-HA (Thermo Fisher Scientific, 88836, RRID: AB_2749815) magnetic beads were rinsed three times in lysis buffer, using 15 μL of beads for every harvested plate. A 50 μL aliquot of the cell lysate was collected as input and the remaining supernatant was incubated with the beads for two hours at 4°C with end-to-end rotation. The immunoprecipitants were washed three times with the same lysis buffer and the cell lysates/immunoprecipitants were resuspended in Tris-Glycine SDS sample buffer (Thermo Fisher Scientific, LC2676) containing 10% 2-mercaptoethanol. Protein was eluted by heating at 95°C for 4 minutes before analysis of protein content by immunoblotting.

### Immunoblotting

For immunoprecipitation experiments, the collected samples were loaded directly for immunoblotting. For measuring steady-state abundance changes, cultured cells were lysed using 1x RIPA buffer (Boston BioProducts, BP-115X) supplemented with 1x protease and phosphatase inhibitor cocktail (Thermo Fisher Scientific, 78441) for 15 minutes at 4°C. Samples were centrifuged at 21,000xg for 15 minutes at 4°C and protein concentration was normalized using a BCA assay (Thermo Fisher Scientific, 23225). Clarified supernatants were resuspended in Tris-Glycine SDS sample buffer (Thermo Fisher Scientific, LC2676) containing 10% 2-mercaptoethanol. Samples were loaded into 4–12% Tris-Glycine 15-well pre-cast gels (Thermo Fisher Scientific, XP04125BOX) and electrophoresis was run in 1x Tris-Glycine SDS running buffer (Thermo Fisher Scientific, LC2675–4) at a constant 165–180 volts until the molecular weight ladder (Thermo Fisher Scientific, 26619) ran to the bottom. The protein within the gel was transferred to a 0.2 μm nitrocellulose membrane (BioRad, 170–4158) using the Trans-Blot Turbo Transfer System (BioRad). Nitrocellulose membranes were then blocked using 5% milk (LabScientific, M-0842) diluted in 1x TBST (CST, 9997S) for at least 30 minutes at room temperature with gentle rocking. Primary antibodies were then diluted directly in the blocking solution at a 1:1000 dilution and incubated overnight at 4°C with gentle rocking. The following primary antibodies were used: rabbit anti-EGR1 (CST, 4153, RRID: AB_2097038), rabbit anti-FosB (CST, 2251, RRID: AB_2106903), rabbit anti-c-Fos (in house) (69), rabbit anti-NR4A1 (in house, warning has high background), rabbit anti-Midnolin (Proteintech, 18939–1-AP, RRID: AB_2878569), rabbit anti-PSMD2 (CST, 25430, RRID: AB_2798903), rabbit anti-PSMA2 (CST, 2455, RRID: AB_2171400), rabbit anti-HA (CST, 3724, RRID: AB_1549585), rabbit anti-FLAG (CST, 14793, RRID: AB_2572291), rabbit anti-mTOR (CST, 2983, RRID: AB_2105622), rabbit anti-Actin (CST, 4970, RRID: AB_2223172), rabbit anti-GAPDH (CST, 5174, RRID: AB_10622025), rabbit anti-CBX4 (CST, 30559, RRID: AB_2798991), rabbit anti-CBX8 (CST, 14696, RRID: AB_2687589), rabbit anti-ATF2 (CST, 35031, RRID: AB_2799069), rabbit anti-ATF3 (CST, 33593, RRID: AB_2799039), rabbit anti-IRF1 (CST, 8478, RRID: AB_10949108), rabbit anti-RelB (CST, 4922, RRID: AB_2179173), rabbit anti-STAT3 (CST, 9139, RRID: AB_331757), rabbit anti-SPINDOC (Sigma, HPA040128, RRID: AB_10673027), rabbit anti-XRCC1 (CST, 2735, RRID: AB_2218471), rabbit anti-CITED1 (Proteintech, 26999–1-AP, RRID: AB_2880718), rabbit anti-SOX12 (Proteintech, 23939–1-AP, RRID: AB_2879368), rabbit anti-FOXP3 (CST, 5298, RRID: AB_10839127), rabbit anti-c-Myc (CST, 5605, RRID: AB_1903938), rabbit anti-p27 (CST, 3686, RRID: AB_2077850), rabbit anti-Ubiquitin (CST, 43124, RRID: AB_2799235), and rabbit anti-IRF4 (CST, 4299, RRID: AB_10547141).

After overnight incubation, the blots were rinsed four times quickly and three additional times for longer, 10-minute incubations using 1x TBST. After rinsing, the blots were incubated with 5% milk in 1x TBST and the following secondary antibodies were applied directly at a 1:2000 dilution: anti-rabbit IgG, HRP-linked (CST, 7074, RRID: AB_2099233) or anti-mouse IgG, HRP-linked (CST, 7076, RRID: AB_330924). The blots were incubated in secondary antibody for 1 hour at room temperature with gentle rocking before rinsing as done for the primary antibody. The blots were exposed to either Pierce ECL western blotting substrate (Thermo Fisher Scientific, 32106) for strong antibodies or highly abundant protein, or Immobilon western chemiluminescent HRP substrate (Sigma, WBKLS0500) for weaker antibodies or less abundant proteins. All immunoblotting data was collected using high sensitivity autoradiography film (Denville Scientific, E3218).

Note: The midnolin antibody (Proteintech, 18939–1-AP) has limitations that are important for readers to consider. The protein levels of endogenous midnolin appear quite low in most cell types and this antibody contains too much background (non-specific bands) to robustly detect endogenous midnolin protein from cell lysates at steady state. However, endogenous midnolin protein can be detected with this antibody if the cells are pre-treated for a few hours with 10 μM MG132 or if the cells are overexpressing midnolin (fig. S11B).

### Mass spectrometry of endogenous midnolin immunoprecipitants

HEK-293T cells expressing endogenous 3xHA-tagged midnolin were cultured to 90% confluency in 5 15 cm plates per condition and unedited wild-type HEK-293T cells were cultured in 5 15 cm plates. The knock-in cells were treated was DMSO or 10 μM MG132 for 6 hours while the unedited wild-type HEK-293T served as background and were treated with 10 μM MG132 for 6 hours. An anti-HA immunoprecipitation was performed using the same lysis conditions and protocol as described in the immunoprecipitation section of the methods. After the final wash, the beads were resuspended in 100 μL of 50 mM Tris pH 8.5 containing 5% SDS and the samples were heated at 95°C for 5 minutes to elute the proteins.

Eluted proteins were then digested using trypsin on S-Trap Micro columns (Protifi, C02-micro-10) following the manufacturer’s protocol. Specifically, proteins were first reduced using 5 mM TCEP for 15 minutes at 55°C and were subsequently alkylated with 20 mM iodoacetamide for 30 minutes in the dark at room temperature. After alkylation, the samples were acidified using phosphoric acid to a final concentration of 2.5% (v/v) and 10 volumes of 100 mM Tris, pH 7.55 in 90% methanol/10% water were added to the samples to dilute the protein. This solution was then passed through S-Trap column by centrifuging for 30 seconds at 4,000xg. Multiple rounds of centrifugation were needed to load the entirety of one sample onto one column. Once the protein was trapped, the column was rinsed three times using 100 mM Tris, pH 7.55 in 90% methanol/10% water, followed by a dry spin, before adding 2 μg of trypsin suspended in 20 μL 50 mM ammonium bicarbonate, pH 8. Columns were kept overnight at 37°C in a humid environment. After digestion, the peptides on the column were eluted by centrifuging three times for 1 minute at 4,000xg using three buffers applied sequentially: first 40 μL ammonium bicarbonate pH 8, second 40 μL 0.2% formic acid in water, and third 40 μL 50% acetonitrile in water. The pooled peptides were dried under reduced pressure using a SpeedVac and were resuspended in 30 μL 0.1% formic acid in water. LC-MS/MS data were acquired as reported previously (70) by injecting 10 μL of resuspended peptide sample.

A protein database consisting of the Human UniProt SwissProt proteome (downloaded on November 13^th^, 2022) was used to identify proteins that co-immunoprecipitated with endogenous 3xHA-midnolin. Specifically, the FragPipe graphical user interface (v18.0) was used to search the data using the MSFragger search engine and to perform post-processing of the search results. The following parameters were used in the search. Tryptic peptides with a maximum of two missed cleavages were considered. Additionally, carbamidomethylation of cysteine was set as a fixed modification, and oxidation of methionine was allowed as a variable modification, with a maximum of four variable modifications per peptide. The allowed mass tolerances were 10 ppm for precursor ions and 0.04 Da for product ions. Peptide hits were filtered to a false discovery rate of 1% using PeptideProphet as implemented in FragPipe.

### Immunofluorescence

For experiments in fig. S5, A and B, 400,000 HEK-293T cells with indicated genetic background were plated on Poly-D-Lysine coated coverslips (TED PELLA, Inc.). On the following day, indicated treatments with DMSO, MG132, or PMA were performed for 6 hours before collection. Culture media was aspirated, and cells were washed with PBS once before fixation by 4% paraformaldehyde (PFA) in PBS for 15 minutes at room temperature. After three PBS washes, cells were permeabilized with 0.05% Triton in PBS for 5 minutes at room temperature. Cells were washed with PBS three times and placed in immunofluorescence blocking buffer (LI-COR, 927–70001) for 45 minutes at room temperature. Primary antibodies mouse anti-FLAG (Sigma, F1804, RRID: AB_262044) and rabbit anti-HA (CST, 3724, RRID: AB_1549585) were diluted 1: 400 in the blocking buffer and added on top of coverslips with cells for an overnight incubation at 4°C. After being rinsed three times with PBS, the cells were incubated in the dark at room temperature with secondary antibodies (1:500 dilution) and 8 μM Hoechst 33342 dye (Thermo Fisher Scientific, H3570), both diluted in blocking buffer, for 1 hour. Coverslips were rinsed thoroughly using PBS and were mounted on glass slides using ProLong gold antifade mountant (Thermo Fisher Scientific, P10144).

An Alexa 488-conjugated secondary antibody was utilized for the FLAG (Thermo Fisher Scientific, A-11001, RRID: AB_2534069) and HA (Thermo Fisher Scientific, A-11008, RRID: AB_143165) staining in fig. S5, A and B, the excitation wavelength was 488 nm. The excitation wavelength of the Hoechst nucleus-staining dye was 405 nm.

Image acquisition was done by a Zeiss AxioVert200M microscope with a 100X oil immersion objective as well as a Yokogawa CSU-22 spinning disk confocal head with a Borealis modification (Spectral Applied Research / Andor) along with a Hamamatsu ORCA-ER CCD camera. The image acquisition and hardware were controlled by the MetaMorph software package (Molecular Devices). The excitation lasers utilized to capture the images were 405 nm and 488 nm.

### RNA extraction and qPCR

Total RNA was extracted from cells with the RNeasy Plus Mini Kit (Qiagen, 74134) and cDNA was generated from freshly extracted RNA using the iScript cDNA Synthesis Kit (BioRad, 1708891) following the manufacturer instructions for both kits. Specifically, 250 ng of RNA was used for a 20 μL reaction to generate the cDNA. Platinum SYBR Green qPCR Supermix-UDG (Thermo Fisher Scientific, 11733038) and 2 μL of cDNA was used for qPCR reactions. Specifically, master mixes were prepared to contain 10 μL of SYBR, 7.5 μL of water, and 0.5 μL of 40x primers per 20 μL qPCR reaction. A Quantstudio 6 Pro (Thermo Fisher Scientific) was used to run the qPCR reactions. The following intercalating pre-mixed qPCR primers were obtained from IDT:

Mouse MIDN (Mm.PT.58.10544931):

GCGTCAACTTGCTCCCAT

AACGCCTCAAAGTACCCAAG

Mouse EGR1 (Mm.PT.58.29064929):

GATAACTCGTCTCCACCATCG

AGCGCCTTCAATCCTCAAG

Mouse c-Fos (Mm.PT.58.29977214):

GGCACTAGAGACGGACAGAT

ACAGCCTTTCCTACTACCATTC

Mouse FosB (Mm.PT.58.10990878):

AGAGACACTTACCCCAGAAGA

GCTCTGCCTTTTCCTCTTCA

Mouse Actin (Mm.PT.39a.22214843.g):

GACTCATCGTACTCCTGCTTG

GATTACTGCTCTGGCTCCTAG

Mouse mTOR (Mm.PT.58.28403918):

TGCATCACTCGTTCATCCTG

AAGTCATCACATCCAAGCAGA

Changes in mRNA levels were determined by subtracting the Cq values generated during the qPCR between the gene of interest and the control to yield a ΔCq value. Data were then normalized to the indicated control condition by substracting the ΔCq values by the average ΔCq of the indicated control condition to generate the ΔΔCq. Plotted in graphs are 2^(-ΔΔCq) from three biological replicates and the following statistical tests were used.

Figure 2, E and F: Data were analyzed using an ordinary one-way ANOVA followed by Tukey’s multiple comparisons test where **** represents p < 0.0001.

Figure S1 D: Data were analyzed using a two-way ANOVA followed by Šidák’s multiple comparisons test where ns is not significant and *** represents a p < 0.001.

### Computational identification of substrate β strands

#### AlphaFold multimer predictions

To identify β strands within hits identified in the ORFeome GPS screen, genes with ΔPSI < −0.5 were taken (n=508) and the longest sequence across corresponding protein accession IDs (either NCBI Reference Sequence or Ensembl ID) was used as the input sequence for downstream steps (as barcodes from the screen were grouped at the gene level but could represent multiple isoforms). These sequences were individually paired with the MIDN sequence (UniProtKB: Q504T8) as a two-sequence FASTA file input into AlphaFold (v2.2.0) for multimer prediction with default reference databases specified as in (26) and max_template_date=2022–01-01. Any selenocysteines were recoded as cysteines and three substrates (ACSBG2, ACSS2, and RIMBP3) that failed MSA using the default settings were rerun successfully by replacing the UniClust30_2018_08 database with UniRef30_2022_02.

### Identification of substrate β strands within Midnolin β sheet

The 25 ranked PDB models from each AlphaFold run with MIDN and one of the substrates were then processed by a custom Python script to identify PDB models that folded a linear stretch of the substrate into β strand conformation placed between β strands of the corresponding MIDN domain. In more detail, a pairwise distance matrix was first computed between each 𝛼-carbon atom in MIDN and each 𝛼-carbon atom in the substrate as

$$D_{i,j}=\sqrt{(x_{i}-x_{j}{)}^{2}+(y_{i}-y_{j}{)}^{2}+(z_{i}-z_{j}{)}^{2}}$$

where ${\text{x}}_{\text{i}}$,${\text{y}}_{\text{i}}$, and ${\text{z}}_{\text{i}}$ are the coordinates of the i^th^ substrate 𝛼-carbon atom and ${\text{x}}_{\text{j}}$, ${\text{y}}_{\text{j}}$, and ${\text{z}}_{\text{j}}$ are the coordinates of the j^th^ MIDN 𝛼-carbon atom. As most β sheets have inter-strand distances < 5 Å (71), the distance matrix was scanned to identify sequential substrate residues < 5.5 Å from corresponding linear stretches within each adjacent MIDN β strand (ie. ${\text{D}}_{\text{i,j}}<5.5$ for both some sequential set of i with some sequential set of j, where 148 ≤ j ≤157, as well as the same set of i with another sequential set of j, where 279 ≤ j ≤ 286).

Secondary structure assignment for the PDB model was done with the DSSP algorithm (72). Substrate residues satisfying the distance requirements specified above were then retained if they were assigned the extended β strand secondary structure (i.e., “E” coding). As DSSP relies on flanking residues to call secondary structure, the most N- and C-terminal residues are not assigned secondary structure. To avoid excluding them from β strand assignments, they were assigned “E” coding if the adjacent residue had been assigned “E” coding. To catch residues that are part of a β strand, but slightly further from one or both of the MIDN β strands, this set of residues was then expanded by 7 residues in each direction and again only those with extended β strand secondary structure were kept. Finally, the longest contiguous stretch of β strand secondary structure was kept (if any) for final reporting (Data S4).

### Properties of identified substrate β strands

#### Relative disorder comparison

ORFs used in AlphaFold multimer folding alongside midnolin were matched with UniProtKB accessions using UniParc to find identical proteins that had already been folded as monomers in the AlphaFold Protein Structure Database (https://alphafold.ebi.ac.uk/). This yielded existing models for 126/205 substrates predicted to interact with the midnolin Catch domain, for which the predicted local distance difference test (pLDDT) scores were extracted from corresponding PDB files. The pLDDT scores for these residues interacting with the midnolin Catch domain were averaged and compared with the average of the rest of the substrate (paired t-test, p = 2.72×10^−9^) as an approximation of disorder in the original substrate (60), where lower pLDDT scores correspond to increased disorder. In conclusion, the regions that are predicted to interact with the Catch domain are predicted to be unstructured by AlphaFold in the native, midnolin-free state.

#### Amino acid enrichment

For each residue, n, going into the β strand from either the N- or C-terminal side, the overall frequency of each amino acid for β strands of length ≥ 2n (to avoid double counting) was normalized by the background amino acid frequency across substrate sequences. Frequencies were then computed for amino acids preceding and following all β strands, provided the β strand was not the N- or C-terminus of the protein respectively.

#### Hydrophobicity

The relative hydrophobicity of residues comprising the MIDN-interacting β strand was assessed by a two-sided paired t-test between the mean hydrophobicity index (58, 59) (at pH 7) of residues in the β strand with that of residues comprising the rest of the substrate.

#### Identification of MIDN-facing β strand side chains

As β strands within a β sheet make contacts with each other through backbone interactions, where side chains alternatingly project above and below the plane of the β sheet, residues composing the MIDN-interacting β strand can be parsed into those facing towards or away from the MIDN Catch domain. Side chains of the substrate β strand facing MIDN Catch domain were identified by first generating another distance matrix as before, but between each 𝛼-carbon atom in MIDN and each 𝛽-carbon atom in the substrate. For each residue in the identified substrate β strand, those with 𝛽-carbon distance to MIDN Catch domain (approximated by 𝛼-carbon position for MIDN isoleucine residue 309) less than their 𝛼-carbon distance were annotated as MIDN-facing. Because glycine lacks a 𝛽-carbon and residues at the ends of β strands may have more rotational variability, but side chain orientations along the β strand should alternate between facing towards or away from the MIDN Catch domain, a filter was then applied to ask whether the identified MIDN-facing side chains better matched either the set of odd or set of even residues and MIDN-facing side chains were then annotated as that set. Properties such as relative frequency and hydrophobicity were then computed for inward- and outward-facing side chains.

## Supplementary Material

Supplemental data and figures

Data 1 EGRI and FosB genome wide CRISPR screends MAGeCK analysis

Data S2 Midnolin GPS ORFeome screen

Data S3 Mass Spectrometry results from endogenous midnolin immunoprecipitation

Data S4 Summary of AlphaFold predicted beta strand degrons

## Data Availability

Any reagents that are unique to this study will be made available upon request. Request for reagents or any additional information necessary to reanalyze the data reported in this paper should be directed to and will be fulfilled by the corresponding authors, Stephen J. Elledge and Michael E. Greenberg. The raw DNA sequencing data, mass spectrometry data, and PDB files for the midnolin-substrate AlphaFold predictions were deposited to Dryad and can be accessed at DOI 10.5061/dryad.m905qfv6g (73).
