# Supplementary material for: The midnolin-proteasome pathway catches proteins for ubiquitination-independent degradation
Source: Science. Author manuscript; Available in PMC 2023 Oct 31. (PMC10617673; doi:10.1126/science.adh5021)
Supplement: Supplemental data and figures [file NIHMS1934122-supplement-Supplemental_data_and_figures.docx]

**The midnolin-proteasome pathway catches proteins for ubiquitination-independent degradation**

Xin Gu^†^, Christopher Nardone^†^, Nolan Kamitaki, Aoyue Mao, Stephen J. Elledge^*^, Michael E. Greenberg^*^

†These authors contributed equally to the work

*Correspondence to: Stephen J. Elledge, [selledge@genetics.med.harvard.edu](mailto:selledge@genetics.med.harvard.edu) and Michael E. Greenberg, [michael_greenberg@hms.harvard.edu](mailto:michael_greenberg@hms.harvard.edu)

**This PDF file includes:**

Figs. S1 to S11

Captions for Data S1 to S4

**Other Supplementary Materials for this manuscript include the following:**

Data S1. EGR1 and FosB genome-wide CRISPR-Cas9 screens MAGeCK analysis

Data S2. Midnolin GPS ORFeome screen results

Data S3. Mass spectrometry results from endogenous midnolin immunoprecipitation

Data S4. Summary of AlphaFold predicted β strand degrons

**Fig. S1. Validation of the CRISPR/Cas9 screens and mRNA expression changes.** (**A**) GPS reporters for EGR1, NR4A1, and c-Fos were stably expressed in HEK-293T cells and BFP or midnolin co-expressing BFP were transiently transfected before analyzing the GFP/DsRed ratio by flow cytometry. (**B**) Similar assay as (A) but gating the analysis for increasing levels of BFP (midnolin) for the GPS FosB reporter. (**C**) Similar assay as (A) for GPS reporters of negative control nuclear proteins such as ATF2, CREB3, and CREB5. (**D**) Midnolin overexpression does not grossly affect the mRNA levels of IEGs. qPCR analysis for mRNA levels of the indicated genes from primary mouse cortical neurons stably overexpressing a control BFP or midnolin co-expressing BFP that were KCl stimulated for the indicated time points. Error bars represent the standard deviation from three biological replicates. Data were analyzed using a two-way ANOVA followed by Šidák’s multiple comparisons test where ns is not significant and *** represents a p < 0.001. (**E**) RiboTag immunoprecipitation followed by RNA seq data extracted from Mardinly et al., Nature 2016, highlighting *MIDN* and various IEGs are induced by light stimuli in the mouse visual cortex (*11*).

**Fig. S2. Validation of the midnolin GPS ORFeome screen.** (**A**) Validation of the GPS ORFeome screen showing the degradation of many putative midnolin substrates upon midnolin overexpression. A control BFP or midnolin co-expressing BFP were transfected into *MIDN* KO HEK-293T cells stably expressing the indicated GPS reporters before analyzing the GFP/DsRed ratio by flow cytometry. These proteins were extracted as possible midnolin substrates based on the GPS ORFeome screen (Data S2).

**Fig. S3. Midnolin does not require active ubiquitin E1 enzymes to function.** (**A**) The BioPlex dataset reveals IEG proteins and the proteasomal component PSMD2 co-immunoprecipitate midnolin from HEK-293T cells (*17*). (**B**) An active ubiquitin E1 enzyme is not required for midnolin-dependent degradation. *MIDN* KO HEK-293T cells stably expressing the indicated GPS reporters for midnolin substrates were transfected with BFP or midnolin co-expressing BFP. The cells were then treated with DMSO, 10 µM MG132, or 500 nM TAK-243 for 6 hours before analyzing the GFP/DsRed ratio by flow cytometry. c-Myc serves as a negative control because it does not appear to be a midnolin substrate but remains targeted for ubiquitination-dependent degradation.

**Fig. S4. AlphaFold confidence and midnolin conservation.** (**A**) AlphaFold prediction of midnolin (Q504T8-F1) colored by the predicted local distance difference test (pLDDT) as a measure of confidence or disorder (*60*). (**B**) AlphaFold prediction of midnolin (Q504T8-F1) colored by conservation using the ConSurf web server.

**Fig. S5. Midnolin is localized primarily within the nucleus.** (**A**) Midnolin nuclear localization requires the C-terminal α helix. Anti-FLAG immunofluorescence was performed from HEK-293T cells stably expressing the indicated 2xFLAG-tagged midnolin constructs and treated with 10 µM MG132 for 6 hours. The Hoechst dye serves as a DNA/nucleus stain. (**B**) Endogenous midnolin is localized primarily within the nucleus. Anti-HA immunofluorescence was performed from wild-type unedited and knock-in 3xHA-midnolin HEK-293T cells treated with the indicated drugs for 6 hours.

**Fig. S6. Further validation of the midnolin functional domains.** (**A**) Regions with defined secondary structure are crucial for a functional midnolin. The GPS FosB reporter was stably expressed in *MIDN* KO HEK-293T cells and a control BFP or wild-type and mutant versions of midnolin co-expressing BFP were transiently transfected before analyzing the GFP/DsRed ratio by flow cytometry. (**B**) The interaction of midnolin with the proteasome is not affected by inhibitors of the proteasome or E1 ubiquitin-activating enzymes. Immunoblotting was performed from anti-FLAG immunoprecipitants of *MIDN* KO HEK-293T cells stably expressing 2xFLAG-tagged midnolin using a CMV promoter. Cells were treated with 10 µM MG132 or 500 nM TAK-243 for 6 hours. (**C**) The Catch domain is necessary for midnolin to interact with its substrates. Immunoblotting was performed from anti-FLAG immunoprecipitants of NIH/3T3 cells stably expressing the indicated 2xFLAG-tagged midnolin constructs. The cells were serum starved overnight and serum restimulated in the presence and absence of 10 µM MG132 before lysis.

**Fig. S7. Midnolin contains a Catch domain with a hydrophobic core that interacts with substrates.** (**A**) The sequence between Catch1 and Catch2 is not required to interact with substrates or the proteasome. Immunoblotting was performed from anti-FLAG immunoprecipitants of *MIDN* KO HEK-293T cells transfected with 2xFLAG-tagged midnolin constructs. See methods for truncation boundaries. Cells were treated with 10 µM MG132 and 20 ng/mL PMA for 6 hours. (**B**) The sequence between Catch1 and Catch2 is dispensable, while the hydrophobic residues that mediate the Catch1 and Catch2 interaction are required for midnolin to promote substrate degradation. The GPS IRF4 or FosB reporter was stably expressed in *MIDN* KO HEK-293T cells and a control BFP and wild-type or mutant versions of midnolin co-expressing BFP were transiently transfected before analyzing the GFP/DsRed ratio by flow cytometry. (**C**) AlphaFold structure prediction and conservation of the Catch domain reveals a hydrophobic core. All residues within the core of the Catch domain are hydrophobic and the side chains are shown in the conservation figure generated using the ConSurf web server. (**D**) The hydrophobic residues that mediate the Catch1 and Catch2 binding are required for the interaction of midnolin with its substrates. Similar assay as (A). (**E**) The conserved hydrophobic core is necessary for the degradative function of midnolin. Similar assay as (B). The immunoblot shows that all versions of midnolin express relatively evenly.

**Fig. S8. AlphaFold prediction of IRF4 bound to midnolin.** (**A**) AlphaFold structure prediction of IRF4 (AF-Q15306-F1), highlighting in red the unstructured region captured by the midnolin Catch domain. (**B**) AlphaFold structure prediction of midnolin bound to IRF4, highlighting the same unstructured region that now forms a β strand conformation that functions as a midnolin degron. (**C**) The predicted local distance difference test (pLDDT) scores for residues that interact with the midnolin Catch domain were averaged and compared with the average of the rest of the substrate as an approximation of disorder in the original substrate not bound by midnolin (paired t-test, p = 2.72x10^-9^).

**Fig. S9. AlphaFold-predicted β strand regions are necessary for capture by midnolin.** Midnolin requires adopted β strands within substrates to promote degradation. GPS reporters for the indicated AlphaFold midnolin-substrate predictions were stably expressed in *MIDN* KO HEK-293T cells that were transfected with BFP or midnolin co-expressing BFP before analyzing the GFP/DsRed ratio by flow cytometry. (**A**) The Δ β strand is defined by a deletion of residues 245-260 of FOXS1. (**B**) The Δ β strand is defined by a deletion of residues 538-558 of CBX4. (**C**) The Δ β strand is defined by a deletion of residues 277-288 of NeuroD1. (**D**) The Δ β strand is defined by a deletion of residues 314-328 of SPINDOC (C11ORF84).

**Fig. S10. Further validation of AlphaFold-predicted β strand regions captured by midnolin.** (**A**) ΔFosB, a naturally expressed splice isoform, shows resistance to midnolin-promoted degradation. A BFP control or midnolin co-expressing BFP were transfected into HEK-293T cells stably expressing the indicated FosB GPS reporters before analyzing the GFP/DsRed ratio by flow cytometry. See methods for more details regarding the truncation boundaries. (**B**) FosB contains multiple regions for midnolin capture. Similar assay as (A). ΔFosB is defined by truncation of the last 101 amino acids, while ΔN β strand is defined by a deletion of residues 67-75 of FosB. (**C**) Related to Fig. 6E. The hydrophobicity of residues comprising the β strand was determined by a two-sided paired t-test (p = 2.1x10^-69^) between the average hydrophobicity index at pH 7 of residues within the β strand, with that of residues comprising the rest of the substrate. (**D**) Similar analysis as (C) but comparing the β strand residues buried within the Catch domain and the residues pointing outward that are solvent exposed (p = 4.6x10^-28^). (**E**) AlphaFold structure prediction of the midnolin Catch domain bound to EGR1, highlighting the hydrophobic residues of EGR1 that are buried within the core of the Catch domain. (**F**) Hydrophobic β strand residues buried within the Catch domain are required for midnolin capture. Immunoblotting was performed from anti-FLAG immunoprecipitants of 3xHA-midnolin knock-in HEK-293T cells transfected with 2xFLAG-tagged EGR1 constructs. Cells were treated with 10 µM MG132 for 6 hours. (**G**) and (**H**) Regions encompassing predicted β strand(s) are sufficient for midnolin to promote degradation. Similar assay as (A). The IRF4 peptide is defined by the region spanning 192-248; the EGR1 peptide is defined by the region spanning 113-172; the FosB peptide is defined by the region spanning 238-338 amino acids.

**Fig. S11. Validation of midnolin clonal knockout cell lines and Proteintech antibody.** (**A**) Regions flanking the sgRNA cut site within the *MIDN* gene locus were PCR amplified, run on an agarose gel, and the PCR product was sequenced using Next-Generation DNA Sequencing to determine the allele identity. (**B**) Immunoblot from wild-type and *MIDN* knockout clone #1. Cells were pre-treated for 6 hours with 10 µM MG132.

**Data S1. EGR1 and FosB genome-wide CRISPR-Cas9 screens MAGeCK analysis (separate file)**

This file contains the data related to the EGR1 and FosB genome-wide CRISPR-Cas9 screens. The MAGeCK analysis and genome-wide sgRNAs are presented.

**Data S2. Midnolin GPS ORFeome screen results (separate file)**

This file contains the data related to the midnolin GPS ORFeome screen. The individual PSI for each barcode and the collapsed ΔPSI is presented.

**Data S3. Mass spectrometry results from endogenous midnolin immunoprecipitation (separate file)**

This file contains the data related to the mass spectrometry results from endogenous midnolin immunoprecipitation.

**Data S4. Summary of AlphaFold predicted β strand degrons (separate file)**

This file contains a summary of the β strand degrons extracted from the midnolin-substrate AlphaFold predictions based on the GPS ORFeome screen. The amino acid sequence of the β strand “aa”, the three amino acids proceeding the β strand “n3_aa”, the three amino following the β strand “c3_aa”, and the amino acids of the β strand pointing within “aa_in” and outward “aa_out” of the midnolin catch domain are presented.
